# Molecular Characterization of a Multidrug-Resistant *Klebsiella pneumoniae* Strain R46 Isolated from a Rabbit

**DOI:** 10.1155/2019/5459190

**Published:** 2019-08-18

**Authors:** Fei Wu, Yuanyuan Ying, Min Yin, Yi Jiang, Chongyang Wu, Changrui Qian, Qianqian Chen, Kai Shen, Cong Cheng, Licheng Zhu, Kewei Li, Teng Xu, Qiyu Bao, Junwan Lu

**Affiliations:** ^1^School of Laboratory Medicine and Life Sciences/Institute of Biomedical Informatics, Wenzhou Medical University, Wenzhou, Zhejiang 325035, China; ^2^College of Medicine and Health, Lishui University, Lishui 323000, China; ^3^Institute of Translational Medicine, Baotou Central Hospital, Baotou 014040, China

## Abstract

To investigate the mechanisms of multiple resistance and the horizontal transfer of resistance genes in animal pathogens, we characterized the molecular structures of the resistance gene-related sequences in a multidrug-resistant *Klebsiella pneumoniae* strain R46 isolated from a rabbit. Molecular cloning was performed to clone the resistance genes, and minimum inhibitory concentrations (MICs) were measured to determine the resistance characteristics of the cloned genes and related strains. A conjugation experiment was conducted to assess the transferability of the resistance plasmids. Sequencing and comparative genomic methods were used to analyze the structures of the resistance gene-related sequences. The *K. pneumoniae* R46 genome consisted of a chromosome and three resistance plasmids named pR46-27, pR46-42, and pR46-270, respectively. The whole genome encoded 34 antibiotic resistance genes including a newly identified chromosome-encoded florfenicol resistance gene named *mdfA2*. pR46-270, besides encoding 26 antibiotic resistance genes, carried four clusters of heavy metal resistance genes and several virulence-related genes or gene clusters. The plasmid-encoded resistance genes were mostly associated with mobile genetic elements. The plasmid with the most similarity to the *floR* gene-harboring plasmid pR46-27 was pCTXM-2271, a plasmid from *Escherichia coli*. The results of this work demonstrated that the plasmids with multidrug resistance genes were present in animal-derived bacteria and more florfenicol resistance genes such as *mdfA2* could be present in bacterial populations. The resistance genes encoded on the plasmids may spread between the bacteria of different species or genera and cause the resistance dissemination.

## 1. Introduction

*Klebsiella pneumoniae*, a member of the Enterobacteriaceae, is pervasive in the natural environment and benignly colonizes the gastrointestinal tracts of humans and animals. It is an opportunistic pathogen capable of causing a wide range of diseases in humans and different animal species [1]. In animals, infections of the urinary and respiratory tracts and sepsis are the most common clinical manifestations. In addition, *K*. *pneumoniae* species are a well-documented cause of mastitis in cattle [2]; endometritis, cystitis, and liver abscess in horses; tracheitis and wounds in birds; cystitis, phlebitis, and otitis externa in dogs; mastitis and wounds in cows; and cystitis in cats, among others [3].

Notably, the prevalence of antibiotic resistance is increasing among Enterobacteriaceae, including *K. pneumoniae* strains isolated from animals [1]. Due to the extensive use of antibiotics in humans, veterinary medicine, and agricultural practice during the last few decades, the emergence of *K. pneumoniae* strains that harbor various resistance genes has increased considerably. *K. pneumoniae* has acquired increasingly high levels of antimicrobial drug resistance. Many publications have reported that *K. pneumoniae* isolated from animals, such as pigs, chickens, and dogs, carries various resistance genes and shows resistance to a variety of antibiotics, such as aminoglycosides (*aadA*1, *aacC2*, and *aacA*4) [4], colistin (*mcr-1*), amphenicols (*floR*) [5], sulfonamides (*sul1* and *sul2*) [6], quinolones (*qnrA*, *qnrB*, *qnrS*, and *qepA*) [7], and *β*-lactams (CTX-M groups) [8]. Multidrug-resistant (MDR), extensively drug-resistant (XDR) [9], and pandrug-resistant (PDR) [10] isolates of *K. pneumoniae* have been reported worldwide.

The antibiotic resistance, especially multiple-drug resistance bacteria of different species in both human and animal pathogens, becomes an increasing global problem. The multidrug resistance in the microbial organisms, in particular the Enterobacteriaceae, is often due to the acquisition of resistance genes from a shared pool [11]. The resistance genes in this pool appeared to have been captured from the chromosomes of various species by the mobile genetic elements, such as insertion sequences (IS) [12], transposons [13], and integrons [14]. A great number of genotypes (subgenotypes) of the antibiotic resistance genes have been found encoded on the mobile genetic elements. These mobile genetic elements can translocate between different bacterial DNA molecules, such as chromosomes and plasmids, especially the conjugative plasmids, and can be transferred between different bacterial species or genera through horizontal gene transfer, causing resistance dissemination [15].

With the widespread use of antimicrobial agents and detergents without effective supervision and the greater incidence of animal-caused foodborne illnesses, animals, especially those in agricultural practice, may become potential reservoirs for the dissemination of antimicrobial resistance. In this work, to investigate the resistance characteristics of *K. pneumoniae* isolated from animals, we used molecular cloning and comparative genomic methods to identify resistance gene profiles and to assess the horizontal transfer of resistance genes in a *K. pneumoniae* strain isolated from a rabbit in a farm in Wenzhou, South China.

## 2. Materials and Methods

### 2.1. Bacterial Isolation and Identification

*K. pneumoniae* R46 was isolated on a MacConkey agar (MCA) plate with florfenicol (8 mg/L) from an anal swab sample of a rabbit from a farm in Wenzhou, South China. Species identification was conducted with the Vitek-60 microorganism auto-analysis system (BioMerieux Corporate, Lyon, France). Further verification was performed by using homology comparisons of the 16S ribosomal RNA gene and the whole genome sequence from NCBI nucleotide database (http://www.ncbi.nlm.nih.gov) via BLASTN software. Multilocus sequence typing (MLST) was performed by analyzing the housekeeping genes *gapA*, *infB*, *mdh*, *pgi*, *phoE*, *rpoB*, and *tonB* [16] via the online service MLST-1.8 (https://cge.cbs.dtu.dk/services/MLST/) [17].

### 2.2. Antimicrobial Susceptibility Testing

The agar dilution method was used to determine the minimum inhibitory concentrations (MICs) of the antibiotics against bacteria in accordance with the guidelines of the Clinical and Laboratory Standards Institute (CLSI document M100-S28, 2017) and European Committee on Antimicrobial Susceptibility Testing (EUCAST 2017). All tests were carried out in triplicate. Briefly, each isolate was diluted in the logarithmic growth period with 0.9% saline to achieve a 0.5 McFarland standard suspension, equivalent to an inoculum with 5^∗^10^5^ CFU/mL. Suspensions of approximately 5 *μ*L were plated on Mueller-Hinton (MH) agar plates (with a series of antibiotic concentrations between 0.0625 and 1,024 mg/L). The inoculated plates were then incubated for 18 hours at 37°C in a constant-temperature incubator. The MIC was defined as the lowest antibiotic concentration showing no colony growth. The commercially available *E. coli* strain ATCC 25922 served to ensure the quality of the plates, and *E. coli* DH5*α* carrying an empty cloning vector was used for comparison.

### 2.3. Cloning of *K. pneumoniae* R46 Resistance Genes

The genomic DNA of *K. pneumoniae* R46 was extracted using an AxyPrep Bacterial Genomic DNA Miniprep Kit (Axygen Scientific, Union City, CA, USA). To clone resistance genes, the primers of the ORFs with their upstream promotor regions were designed by using Primer Premier 5.0 and were synthesized by Shanghai Sunny Biotechnology Co. Ltd. (Shanghai, China) (Table 1). A standard protocol for PCR with PrimeSTAR HS DNA Polymerase (TaKaRa, Dalian, China) was performed under the following conditions: an initial cycle of 94°C for 1 min, followed by 35 cycles of 10 s at 94°C, 15 s at a specific annealing temperature, and then an extension step of 1 min/kb at 72°C, with a final extension step of 10 min at 72°C (Table 1). The PCR products were cloned into the pMD™19-T vector (TaKaRa, Dalian, China) (Table 2). The resulting recombinant plasmids were transformed into *E. coli* DH5*α* using the calcium chloride method, and bacterial colonies were grown on Luria-Bertani agar plates supplemented with ampicillin (100 mg/L). The recombinant plasmids were isolated and PCR-amplified to confirm the sizes of the inserted fragments. The cloned fragments from the transformants were further characterized by Sanger sequencing (ABI3730 Analyzer, USA) and compared to reference resistance genes using the BLASTN program.

### 2.4. Conjugation Experiment

A conjugation experiment was carried out by biparental mating on sterile nitrocellulose filters, and rifampin-resistant *E. coli* C600 (EC600) was used as the recipient. Overnight cultures of the donor strain *K. pneumoniae* R46 and the recipient strain EC600 were mixed together in 3 mL of LB broth, harvested, and resuspended in 80 *μ*L of LB. The mixture was spotted on a 1 cm^2^ filter membrane, placed on an LB plate, and then incubated for mating at 37°C for 12-18 hours. The transconjugants were selected on two kinds of Mueller-Hinton agar plates: one containing 1,024 mg/L rifampin and 32 mg/L florfenicol and the other containing 1,024 mg/L rifampin and 32 mg/L chloramphenicol. The plasmid DNA of the transconjugant was extracted and analyzed by agarose gel electrophoresis and compared to the plasmid profile of *K. pneumoniae* R46. PCR analysis of the genes encoded in the plasmid was performed to further verify the plasmid in the transconjugants.

### 2.5. Sequencing and Annotations of the *K. pneumoniae* R46 Genome

Genomic DNA was extracted from *K. pneumoniae* R46 as mentioned above and sequenced with Pacific Biosciences sequencers at Annoroad Gene Technology Co. LTD (Beijing, China). In addition, a paired-end library with 300 bp insert sizes was constructed and sequenced from both ends with Illumina HiSeq-2500. Pacific Biosciences sequencing reads of approximately 10-20 kb in length were assembled by Canu v1.2 [18]. The Illumina reads were then mapped onto the assembled contigs to correct the primary assembly by using bwa0.7.13, samtools1.3, and GenomeAnalysisTK2.3.9 [19, 20]. Glimmer3.02 software with default parameters was used to predict potential open reading frames (ORFs) [21]. ORF annotations were determined by performing BLASTX comparisons with the NCBI nonredundant protein database. Comparisons of nucleotide sequences and amino acid sequences were performed by BLASTN and BLASTP, respectively [22]. BLASTP was applied to compare amino acid sequences with those in the Antibiotic Resistance Genes Database (ARDB). A map of the plasmid with GC content and GC skew was drawn with the online CGView Server (http://stothard.afns.ualberta.ca/cgview_server/) and local GView 1.7 with a visual interface [23]. The rRNA gene sequences were annotated by the online tool RNAmmer (http://www.cbs.dtu.dk/services/RNAmmer/) [24], and the tRNA sequences were annotated by the online tool tRNAscan-SE 2.0 (http://lowelab.ucsc.edu/tRNAscan-SE/) [25]. Promoter sites were determined by using Soft Berry BPROM software (http://linux1.softberry.com/berry.phtml?topic=bprom&group=programs&subgroup=gfindb). The plasmid sequences used in this study were downloaded from the NCBI nucleotide database (http://www.ncbi.nlm.nih.gov).

### 2.6. Comparative Genomic Analysis

Sequences containing resistance genes were obtained from the NCBI nucleotide database by the BLASTN program using the resistance gene sequences of *K. pneumoniae* R46 as the query. The resulting sequences were filtered, and only sequences containing resistance genes were retained. CD-HIT was used to cluster the retained sequences using the genome sequence of *K. pneumoniae* R46 as the reference with identity of 90% and coverage of 85% [26]. The sequence sharing the greatest similarity to the other sequences in each cluster was chosen as the candidate for ortholog analysis. Orthologous groups of the genes from the candidate sequences were identified using BLASTP [22]. Sequence retrieval, statistical analysis, and other bioinformatic tools used in this study were applied with Perl and Bioperl scripts (http://www.perl.org/).

### 2.7. Nucleotide Accession Numbers

The sequences are now available in the NCBI nucleotide database. The accession numbers for the chromosome and the plasmids are CP035777, CP035774, CP035775, and CP035776, respectively.

## 3. Results

### 3.1. Isolation and Identification of *K. pneumoniae* R46

*K. pneumoniae* R46 is a gram-negative bacillus with small, nonhemolytic, gray, and mucoid colonies after overnight culture on blood agar plates. Whole-genome comparative analysis combined with 16S ribosomal RNA gene homology analysis showed that the most closely related species were three *Klebsiella pneumoniae* strains: *Klebsiella pneumoniae* strain INF042 (CP024542), *Klebsiella pneumoniae* strain INF059 (CP024545), and *Klebsiella pneumoniae* strain KSB1_7J (CP024548) all with coverages of 99% and identities of 99%. MLST of seven housekeeping genes from the *K. pneumoniae* R46 genome showed optimum matching to the same housekeeping genes of the *K. pneumoniae* ST37 genome, with identities of 100.00%, 100.00%, 100.00%, 99.77%, 100.00%, 100.00%, and 100.00%, respectively. Finally, we classified the strain into the genus *K. pneumoniae* and named it *K. pneumoniae* R46 with MLST type ST37.

### 3.2. General Features of the *K. pneumoniae* R46 Genome

**Figure 1:**
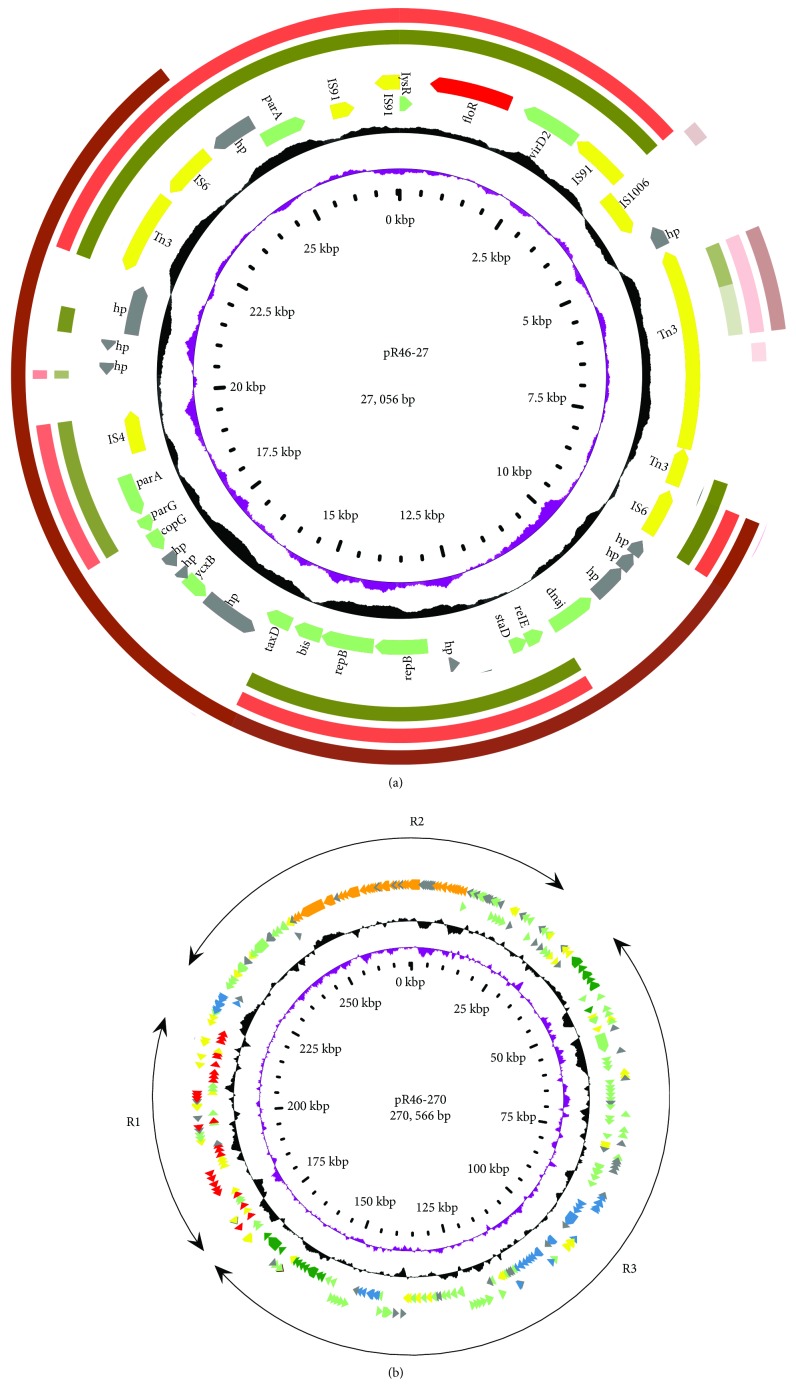
Circular maps of the plasmids pR46-27 (a) and pR46-270 (b). (a, b) Counting from the center toward the outside: (1) the inner most circle shows the position in kb. (2) GC skew (G-C/G+C), with a positive GC skew toward the outside and a negative GC skew toward the inside. (3) GC content, with an average of 50%, whereby a G+C content of more than 50% is shown toward the outside, otherwise, inward. (4) Genes encoded in the leading strands (outwards) or lagging strands (inwards). Genes with different functions are shown in different colors: red: drug resistance; yellow: mobile genetic elements; orange: transfer conjugation; green: virulence; blue: heavy metal resistance; gray: genes with unknown functions; light green: genes with other functions. (a) The plasmid pR46-27 was used as the reference genome and compared to the sequences of pCTXM-2771 (MF589339), pACN001-A (KC853434), and p160070-CTXM (MG288677). (b) R1: multidrug resistance region; R2: transfer and maintenance region; R3: heavy metal resistance and virulence region.

The *K. pneumoniae* R46 genome consists of a 5.12 Mb chromosome encoding 4,701 ORFs and three plasmids, namely, pR46-27, pR46-42, and pR46-270 (Table 3, Figures 1(a) and 1(b)). A total of 34 antibiotic resistance genes were identified in the whole genome, of which five, one, two, and twenty-six were encoded in the chromosome and in the plasmids pR46-27, pR46-42, and pR46-270, respectively (Table 4). The resistance genes were mainly related to the antibiotics streptomycin, aminoglycosides, *β*-lactams, and amphenicols. Moreover, four clusters of heavy metal resistance genes (operons related to silver, copper, arsenic, and mercury resistance) were identified in pR46-270 (Table 4). Interestingly, pR46-27 and pR46-42 belonged to the same incompatibility group IncX, with the X1 and X4 replicons, respectively, while pR46-270 was a multireplicon plasmid with three replicons: Q, FII(k), and FIB(k).

### 3.3. MIC Results for *K. pneumoniae* R46 and the Cloned Resistance Genes

The MIC results for sixteen antibiotics showed that the wild-type *K. pneumoniae* R46 was resistant to eleven antibiotics (68.75%, 11/16), including florfenicol (>512 mg/L), chloramphenicol (256 mg/L), nalidixic acid (>32 mg/L), neomycin (>8 mg/L), ampicillin (512 mg/L), tetracycline (512 mg/L), tigecycline (4 mg/L), aztreonam (32 mg/L), gentamicin (32 mg/L), streptomycin (>32 mg/L), and kanamycin (512 mg/L). The cloned genes also conferred resistance to their corresponding antibiotics at specific levels (Table 5). Interestingly, the cloned *mdfA2* gene, in addition to conferring resistance to chloramphenicol (32 mg/L), also conferred resistance to florfenicol (32 mg/L) with approximately 4-fold increases in the MIC levels over those of the controls DH5*α* (Table 5). The protein sharing the greatest amino acid sequence identity with MdfA2 was MdfA (85.37%, accession number AFH35853) among the function known proteins in ARDB.

### 3.4. Comparative Analysis of the Sequence Region Containing the *mdfA* Gene

**Figure 2:**
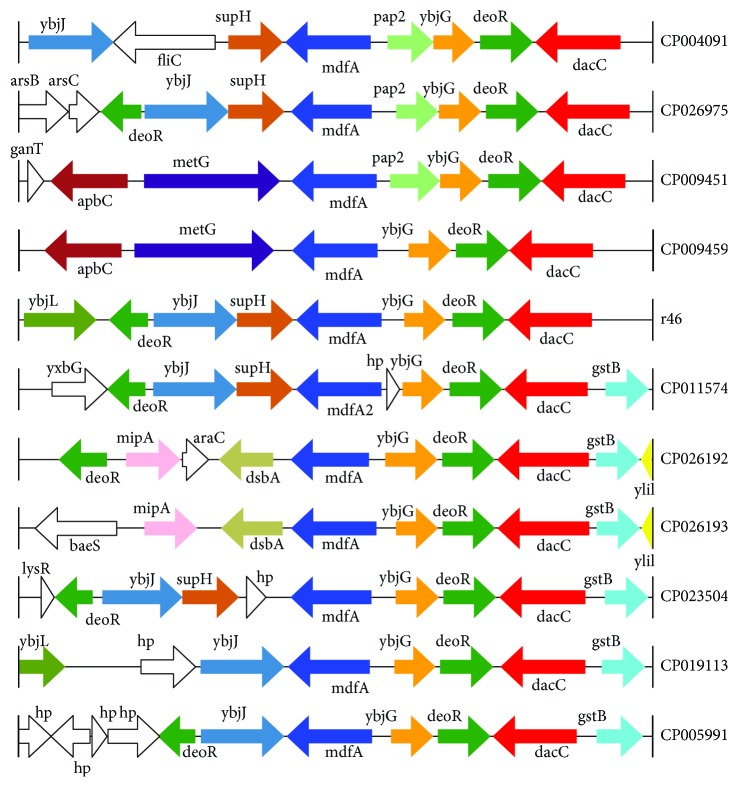
Comparative analysis of the mdfA-related regions of 11 representatives from 980 sequences. The representative sequences were derived from the following bacteria: CP004091 (*C. sakazakii* SP291 CP004091), CP026975 (*E. cloacae* complex bacterium FDAARGOS_77 CP026975), CP009451 (*C. neteri* SSMD04 CP009451), CP009459 (*C. neteri* ND14a CP009459), CP011574 (*K. aerogenes* CAV1320 CP011574), CP026192 (Enterobacteriaceae bacterium ENNIH2 CP026192), CP026193 (Enterobacteriaceae bacterium ENNIH1 CP026193), CP023504 (*C. werkmanii* FDAARGOS_364 CP023504), CP019113 (*Enterobacter* sp. SA187 CP019113), and CP005991 (*Enterobacter sp*. R4-368 CP005991). Homologous genes are shown in the same colors, whereas the white arrows indicate nonhomologous genes.

A set of 979 sequences totaling approximately 9 kb in length with the *mdfA* gene in the center (with ≥79% nucleotide sequence identities and ≥88% sequence coverage) was retrieved from the NCBI nucleotide database. Of the 980 sequences (including one in *K. pneumoniae* R46), most were from *Klebsiella* (28.06%, 275/980), *Escherichia* (32.65%, 320/980), and *Salmonella* (30.71%, 301/980). The sequences were clustered into 53 clusters with a nucleotide sequence identity of 90% and coverage of 85%. The sequences sharing the greatest similarity in each cluster were chosen as candidates for further grouping according to the genes flanking *mdfA.* Finally, 11 groups were obtained. Most of the sequences (92.04%, 902/980) were grouped into 2 groups, group 7 (59.08%, 579/980) and group 11 (32.96%, 323/980), and the *mdfA2*-containing fragment of *K. pneumoniae* R46 was in group 7 ([Supplementary-material supplementary-material-1]). Eleven representative sequences from 11 groups (one from each group) are illustrated according to their accession numbers (Figure 2, [Supplementary-material supplementary-material-1]). The results of this gene neighborhood analysis revealed that a few of the genes downstream of *mdfA* were conserved and a sequence of approximately 4 kb encoding *mdfA*-*ybjG*-*deoR-dacC* was present in 93.06% (912/980) of the sequences. In contrast, a portion of the genes flanking *mdfA* from the same or different sequence groups differed greatly.

The sequence structures next to *mdfA* in the same genus were relatively conserved. Most of the sequences from *Klebsiella* (86.90%, 239/275) were clustered together in cluster 1, nearly all of the sequences from *Escherichia* (98.75%, 316/320) were in cluster 41, and almost all of the sequences from *Salmonella* (99.34%, 299/301) were in cluster 47 ([Supplementary-material supplementary-material-1]). All of the sequences from *Escherichia* (320/320, 100%) in group 5 shared the same gene structure: *apbC*-*metG*-*mdfA*-*ybjG*-*deoR*. Among the 579 sequences in group 3 with the structure *ybjJ*-*supH*-*mdfA*-hp-*ybjG*, 576 were from *Klebsiella* (263) and *Salmonella* (301), representing 95.64% (263/275) of the *Klebsiella* sequences and 100% (301/301) of the *Salmonella* sequences ([Supplementary-material supplementary-material-1]).

### 3.5. Comparative Analysis of the *floR* Gene-Carrying Plasmid pR46-27

The three plasmids sharing the highest nucleotide sequence similarities (coverage > 60%, identities ≥ 99%) with pR46-27 were retrieved from the NCBI nucleotide database. Two of the plasmids, pCTXM-2271 (MF589339, coverage 66%) from *Escherichia coli* 2271 and pACN001-A (KC853434, coverage 64%) from *Escherichia coli* ACN001, harbored *floR* gene, while the other one, p160070-CTXM (MG288677, coverage 63%) from *Klebsiella pneumoniae* F160070, lacked the *floR* gene. pCTXM-2271 is 222 kb in length and encodes 271 ORFs, which is 195 kb and 234 ORFs more than pR46-27. It had 21 similar ORFs to pR46-27, accounting for 56.76% (21/37) of the ORFs in pR46-27 (Figure 1(a)). The pACN001-A plasmid is approximately 18.6 kb in length and shared nearly the same region with pR46-27 as pCTXM-2271. The ORFs of pR46-27 that matched those of the three plasmids included replication genes (*bis* and *repB*), a stabilization gene (*staD*), and partitioning genes (*parA* and *parG*). The *floR*-encoding region, flanked by a pair of IS*91* sequences, had a transposon structure, and the *virD2*-*floR-lysR* gene cluster was conserved in three plasmids, excluding p160070-CTXM which does not harbor the *floR* gene (Figure 1(a)).

### 3.6. The Conjugative Plasmid pR46-270 Carries Multiple Resistance and Virulence Genes

**Figure 3:**
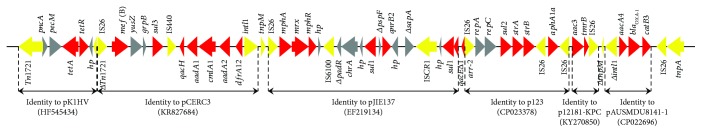
Schematic representation of the MDR region of plasmid pR46-270. Red arrows indicate resistance genes, yellow arrows indicate mobile genetic elements, and gray arrows indicate genes with other functions. Truncated genes are indicated by the “*Δ*” symbol.

Sequence annotation revealed that a conjugative system consisting of 24 *tra* genes, 5 *trb* genes, and a *finO* gene was encoded in a region approximately 36.5 kb in length in pR46-270 (Figure 1(b)). The conjugation experiment confirmed the transferability of pR46-270, facilitating the mobilization of pR46-27 (Table 5). All 26 resistance genes carried by pR46-270 were clustered in a region 47.5 kb in length (Figure 1(b), Figure 3). Interestingly, all three known sulfonamide genes (*sul1* (orf00311 and orf00319), *sul2* (orf00327), and *sul3* (orf00284)) were found to be encoded in one *K. pneumoniae* plasmid for the first time in this study. Comparative genomic analysis demonstrated that the MDR region could be divided into six fragments, and all of the resistance genes were associated with the mobile genetic elements, including two class 1 integrons. The first fragment was a transposon mediated by Tn1721 (5,749 bp in length, between 177.11 and 171.36 kb) encoding two resistance genes, *tetA* and *tetR*, which showed >99% similarity to a fragment in pK1HV from a *K. pneumoniae* strain isolated from a healthy neonate in Vietnam. The second was a class 1 integron (11,770 bp in length, between 177.17 and 188.94 kb) carrying 5 resistance gene cassettes, *dfrA12*, *aadA2*, *cmlA1*, *aadA1*, and *qacH*, that showed >99% nucleotide sequence identity to a fragment in pCERC3 from an *E. coli* strain isolated from a healthy Australian adult. The third was a 13,942 bp fragment (between 189.68 and 203.63 kb) containing seven complete or truncated resistance genes (*mphA*, *mrx*, *mphR*, *sul1*, *qnrB2*, *qacEΔ*1, and *arr-2*) and three insertion sequences (IS, IS*26*, IS*6100*, and IS*CR1*). This fragment shared the highest identity (99%) with a sequence in plasmid pJIE137 from a *K. pneumoniae* strain isolated from a patient in Australia. The fourth fragment was a 7,634 bp sequence (between 204.11 and 211.74 kb) that could be divided into two parts. One part contained a truncated Tn*6029* (containing *repA* and *repC* of the *repABC* operon) and three resistance genes (*sul2* and *strAB*). The other part was a Tn*4352* containing two copies of IS*26* and *aphA1a*. Both parts were similar (>99% identity) to a region in plasmid p123 from an *E. coli* strain isolated from a canine. The fifth fragment, containing IS*26*, *aac3*, and *tmrB*, was a 1,934 bp sequence (between 211.97 and 223.91 kb) that showed >99% similarity to a fragment in plasmid p12181-KPC from a *K. pneumoniae* strain isolated from a patient in China. The last fragment was a 3,374 bp sequence (between 214.38 and 217.75 kb) composed of a class 1 integron with three resistance gene cassettes (*aacA4*, *bla*_OXA-1_, and *catB3*) that showed >99% sequence similarity to a fragment in plasmid pAUSMDU8141-1 from a *Citrobacter farmeri* strain isolated from a patient.

In addition to the multiantibiotic resistance genes, four clusters (operons) of heavy metal resistance genes, including those for silver (*silA*, *silB*, *silC*, *silE*, *silF*, *silR*, and *silS*), copper (*copA*, *copB*, *copC*, *copD*, *pcoE*, *copG*, *pcoR*, and *pcoS*), arsenic (*arsR*, *arsD*, *arsA*, *arsB*, and *arsC*), and mercury (*merA*, *merC*, *merP*, *merE*, *merD*, *merT*, and *merR*), were identified in the plasmid. Moreover, several gene clusters or genes related to virulence were identified in the plasmid, such as two iron uptake systems (*fecABCDEIR* and *iucABCDiutA*), the genes of the *lac* operon (*lacIYZ*), and the fimbrial protein gene *fimK*.

## 4. Discussion

In this work, we analyzed the MDR strain *K. pneumoniae* R46 of ST37 isolated from a rabbit in south China. According to MLST typing, there are more than 3,000 ST types of *K. pneumoniae* identified worldwide (http://bigsdb.pasteur.fr), of which ST37 strains have mostly been reported to be MDR clones. The *K. pneumoniae* ST37 bacterium has largely been isolated from human clinical samples [27, 28]. Recently, ST37 was reported to have been isolated from animals, including companion animals [29] and chickens [30]. *K. pneumoniae* R46 carried 34 resistance genes and showed resistance to a variety of antibiotics, including *β*-lactams, amphenicols, aminoglycosides, and quinolones. In addition, this strain harbored three resistance plasmids, and 76.47% (26/34) of its resistance genes were encoded in a conjugative plasmid, all of the resistance genes were associated with mobile genetic elements. It demonstrated that horizontal gene transfer played an important role in the development of the multidrug resistance of *K. pneumoniae* R46.

*K. pneumoniae* R46 showed a high resistance level to florfenicol (MIC > 512 mg/L). Of the eleven known florfenicol resistance genes, *floR*, *floRv*, *flo_St_*, *optrA*, *pexA*, *fexB*, *fexA*, *cfr*, *cfr(B)*, *cfr(C)*, and *estDL136*, only *floR* was found in the pR46-27 plasmid. Interestingly, an *mdfA* variant, named *mdfA2*, was encoded in the *K. pneumoniae* R46 chromosome and conferred resistance to florfenicol. MdfA has been reported to confer resistance to a variety of antibiotics, such as chloramphenicol [31], ciprofloxacin [32], and fluoroquinolone [33], while its resistance to florfenicol has never been documented.

To date, all *mdfA* variants have been found to be encoded in chromosomes, and no mobile genetic elements have been identified in their neighboring regions, indicating that *mdfA* genes are relatively conserved and might not be carried and transferred by mobile genetic elements. Our comparative genomic analysis demonstrated that the sequences next to the *mdfA* genes in different bacterial genera had different structures, while sequences of the same species or genera, to some extent, showed conserved structures. These observations indicated that sequence rearrangement rarely occurs in the *mdfA*-encoding region.

In addition to antibiotic agents used for human and animal therapy, a large number of other chemical substances with antibacterial activities, such as heavy metals and detergents, are used in human health care and agricultural practices. In addition to encoding 26 antibiotic resistance genes, the pR46-270 plasmid harbored 4 clusters of heavy metal resistance genes (including silver, copper, arsenic, and mercury, a total of 29 genes) and a number of other metal (iron) and virulence-related genes or gene clusters. Associations between heavy metal and antibiotic resistance have been reported previously [34]. Resistance to metals and to antibiotics might be subject to coselection [34]. Metal contamination represents a long-standing, widespread, and recalcitrant selective pressure with both environmental and clinical importance, and it potentially contributes to the maintenance and spread of antibiotic resistance factors.

## 5. Conclusion

Whole-genome sequencing of an animal-derived *K. pneumoniae* isolate harboring three resistance plasmids revealed that in addition to *floR* encoded in the pR46-27 plasmid, a chromosome-encoded *mdfA* variant, named *mdfA2*, also conferred florfenicol resistance. The genome encoded 34 drug resistance genes, of which 26 were encoded in the conjugative plasmid pR46-270. These 26 resistance genes were all associated with mobile genetic elements that shared homologous sequences in different bacterial genera of different origins. Four clusters of heavy metal (silver, copper, arsenic, and mercury) resistance genes and a number of virulence-related genes or gene clusters were also identified in the pR46-270 plasmid. The results of this work demonstrated that the plasmids with multidrug resistance genes were present in animal-derived bacteria and more florfenicol resistance genes such as *mdfA2* could be present in bacterial populations. Active surveillance efforts are imperative for monitoring the prevalence of antibiotic-resistant bacteria, especially florfenicol-resistant strains, in animals around the world.

## Figures and Tables

**Table 1:** Primers used in this study.

Resistance genes	Primer	Sequence (5-3′)^b^	Restriction endonuclease	Vector	Amplicon size (bp)	Annealing temperature (°C)
*catB3* ^a^	P-catB3-F	GGGGTACCACTTACAGGAAACTTGGGGT	*Kpn* I	pMD™19-T	859	58
	P-catB3-R	CGGGATCCTTAGACGGCAAACTCGAGCC	*Bam* HI			

*floR* ^a^	P-floR-F	CGGGATCCGAAGCAAAAGATAATCGGAT	*Bam* HI	pMD™19-T	1412	54
	P-floR-R	CCAAGCTTTTAGACGACTGGCGACTTCT	*Hind* III			

*mdfA2* ^a^	P-mdfA2-F	CGGGATCCTCACATTGCTGAAACATAAACGG	*Bam* HI	pMD™19-T	1400	55
	P-mdfA2-R	CCAAGCTTCTACCCCTGCTGCGAATTGC	*Hind* III			

*qnrB2* ^a^	P-qnrB2-F	CGGGATCCGATTTGACGCATAACCTCAT	*Bam* HI	pMD™19-T	713	55
	P-qnrB2-R	CCAAGCTTCTAGCCAATAATCGCGATGC	*Hind* III			

^a^The primers for ORFs with the predicted promoter regions. ^b^The underlines present the restriction enzyme sites and their protective bases.

**Table 2:** Strains and plasmids used in this study.

Strains and plasmids	Relevant characteristic(s)	Reference or source
R46	A *K. pneumoniae* strain named R46 isolated from a rabbit	This study
DH5*α*	*Escherichia coli* DH5*α* was used as a host for the recombinant plasmid with the cloned resistance gene	Our lab collection
ATCC 25922	*Escherichia coli* ATCC 25922 is an FDA clinical isolate	Our lab collection
pMDTM19-T-ORFs/DH5*α*	DH5*α* carrying the recombinant plasmids pMDTM19-T-ORF (*floR*, *mdfA2*, *qnrB3*, and *catB3*)	This study
*E*. *coli* C600	*E*. *coli* C600 as recipient in the conjugation experiment, RIF^r^	Our lab collection
Plasmids		
pMDTM19-T	Cloning vector for the PCR products of resistance genes of *floR*, *mdfA2*, *qnrB3*, and *catB3*, AMP^r^	This study

^∗^RIF: rifampin; AMP: ampicillin.

**Table 3:** General features of *K. pneumoniae* R46 genome.

	Chromosome	pR46-27	pR46-42	pR46-270
Size (bp)	5,117,042	27,056	42,640	270,566
G+C (%)	57.69	50.08	41.57	51.96
Total opening reading frames	4,701	37	68	307
Known proteins	4,125 (87.75%)	25 (67.57%)	38 (55.88%)	251 (81.76%)
Hypothetical proteins	576 (12.25%)	12 (32.43%)	30 (44.12%)	56 (18.24%)
Protein coding sequence (%)	87.3	82.05	84	83.97
Average ORF length (bp)	950	600	526	740
rRNA operons	1^∗^(16s-23s-5s-5s) 1^∗^(16s-23s-5s) 5^∗^(5s-23s-16s)			
tRNA	87			

**Table 4:** Resistance genes encoded on the *K. pneumoniae* R46 genome.

Genome	Class of resistance genes	Resistance genes
Chromosome	Quinolone	*oqxA*, *oqxB*
	Fosfomycin	*fosA*
	*β*-Lactams	*bla*_SHV-1_
	Amphenicols	*mdfA2*
pR46-27	Amphenicols	*floR*
pR46-42	*β*-Lactams	*bla*_CTX-M-99_, *bla*_CTX-M-14_
pR46-270	*β*-Lactams	*bla*_OXA-1_
	Tetracycline	*tetA*, *tetR*
	Macrolide	*mef*(B), *mphA*, *mrx*, *mphR*
	Sulfonamide	2*sul1*, *sul2*, *sul3*
	Quaternary ammonium compounds	*qacH*, *qacE*Δ1
	Trimethoprim	*dfrA12*
	Quinolone	*qnrB2*
	Rifampin	*arr-2*
	Aminoglycosides	*aacA4*, *aac3*, *aadA1*, *aadA2*, *cmlA1*, *strA*, *strB*, *aphA1a*
	Amphenicols	*catB3*
	Tunicamycin	*tmrB*
	Silver	*silA*, *silB*, *silC*, *silE*, *silF*, *silR*, *silS*
	Copper	*copA*, *copB*, *copC*, *copD*, *pcoE*, *copG*, *pcoR*, *pcoS*
	Arsenic	*arsR*, *arsD*, *arsA*, *arsB*, *arsC*
	Mercury	*merA*, *merC*, *merP*, *merE*, *merD*, *merT*, *merR*

**Table 5:** MICs (*μ*g/mL) for *K. pneumoniae* R46, its cloned genes, and transconjugant (R46/EC600).

Strain	FFC	CHL	NOR	AMK	NAL	NEO^a^	AMP	TCY	TGC^a^	RIF	POL	ATM	GEN	CNX	STR	KAN
ATCC 25922	4	4	0.25	2	2	4	8	1	0.25	4	0.5	<0.5	1	0.5	8	2
*E*. *coli* C600	8	4	4	1	2	>8	4	0.5	2	1,024	0.5	<0.5	8	1	2	1
Transconjugant (R46/EC600)	512	128	8	1	16	>8	512	512	0.25	512	<0.0625	32	32	<0.0625	>32	512
*K. pneumoniae* R46	>512	256	8	4	>32	>8	512	512	4	16	1	32	32	1	>32	512
pMD™19-T-*floR*/DH5*α*	128	64	<0.0625	<0.0625	2	4	512	0.5	0.25	1	0.25	<0.5	1	0.5	<0.5	2
pMD™19-T-*mdfA2*/DH5*α*	32	32	0.25	1	4	4	>512	1	0.25	2	0.25	<0.5	1	0.5	4	2
pMD™19-T-*qnrB2*/DH5*α*	8	8	8	2	16	2	>512	1	0.25	8	0.5	<0.5	1	0.5	4	2
pMD™19-T-*catB3*/DH5*α*	8	8	<0.0625	2	8	4	>512	1	0.25	2	0.25	<0.5	1	0.5	4	2
*E*. *coli* DH5*α*	8	4	<0.0625	0.5	2	2	<1	0.5	0.25	2	0.25	<0.5	1	0.25	2	2

FFC: florfenicol; CHL: chloramphenicol; NOR: norfloxacin; AMK: amikacin; NAL: nalidixic; NEO: neomycin; AMP: ampicillin; TCY: tetracycline; TGC: tigecycline; RIF: rifampin; POL: polymyxin; ATM: aztreonam; GEN: gentamicin; CNX: cefminox; STR: streptomycin; KAN: kanamycin. ^a^For neomycin and tigecycline, we used EUCAST 2017 as the guideline.

## Data Availability

The data related to this paper are deposited in the NCBI database.

## Abbreviations

BLAST: Basic local alignment search tool.

## Supplementary Materials

Supplementary Materials Table S1: 980 sequences containing mdfA gene clustered by CD-HIT with 90% identity and 85% coverage.
