# Supplementary material for: Molecular Characterization of a Multidrug-Resistant Klebsiella pneumoniae Strain R46 Isolated from a Rabbit
Source: Int J Genomics. 2019 Aug 18;2019:5459190. doi: 10.1155/2019/5459190 (PMC6721500; doi:10.1155/2019/5459190)
Supplement: Supplementary Materials — Table S1: 980 sequences containing mdfA gene clustered by CD-HIT with 90% identity and 85% coverage. [file 5459190.f1.doc]

**Table S1. 980 sequences containing *mdfA* gene clustered by CD-HIT with 90% identity and**

**85% coverage**

| Accession number | Cluster | Group | Genus | Detailed information about the bacteria |
| --- | --- | --- | --- | --- |
| AP006725 | 1 | 11 | *Klebsiella* | *Klebsiella pneumoniae* subsp. pneumoniae NTUH-K2044 DNA, complete genome |
| AP014950 | 1 | 11 | *Klebsiella* | *Klebsiella pneumoniae* YH43, complete genome |
| CP000647 | 1 | 11 | *Klebsiella* | *Klebsiella pneumoniae* subsp. pneumoniae MGH 78578, complete genome |
| CP000964 | 1 | 11 | *Klebsiella* | *Klebsiella pneumoniae* 342, complete genome |
| CP001891 | 1 | 11 | *Klebsiella* | *Klebsiella variicola* At-22, complete genome |
| CP002910 | 1 | 11 | *Klebsiella* | *Klebsiella pneumoniae* KCTC 2242, complete genome |
| CP003200 | 1 | 11 | *Klebsiella* | *Klebsiella pneumoniae* subsp. pneumoniae HS11286, complete genome |
| CP003785 | 1 | 11 | *Klebsiella* | *Klebsiella pneumoniae* subsp. pneumoniae 1084, complete genome |
| CP003999 | 1 | 11 | *Klebsiella* | *Klebsiella pneumoniae* subsp. pneumoniae Kp13, complete genome |
| CP006648 | 1 | 11 | *Klebsiella* | *Klebsiella pneumoniae* CG43, complete genome |
| CP006656 | 1 | 11 | *Klebsiella* | *Klebsiella pneumoniae* JM45, complete genome |
| CP006659 | 1 | 11 | *Klebsiella* | *Klebsiella pneumoniae* ATCC BAA-2146 , complete sequence |
| CP006722 | 1 | 11 | *Klebsiella* | *Klebsiella pneumoniae* subsp. pneumoniae 1158, complete genome |
| CP006738 | 1 | 11 | *Klebsiella* | *Klebsiella pneumoniae* HK787, complete genome |
| CP006798 | 1 | 11 | *Klebsiella* | *Klebsiella pneumoniae* subsp. pneumoniae PittNDM01, complete genome |
| CP006918 | 1 | 11 | *Klebsiella* | *Klebsiella pneumoniae* 30684/NJST258_2, complete genome |
| CP006923 | 1 | 11 | *Klebsiella* | *Klebsiella pneumoniae* 30660/NJST258_1, complete genome |
| CP007727 | 1 | 11 | *Klebsiella* | *Klebsiella pneumoniae* subsp. pneumoniae KPNIH10, complete genome |
| CP007731 | 1 | 11 | *Klebsiella* | *Klebsiella pneumoniae* subsp. pneumoniae KPNIH27, complete genome |
| CP008797 | 1 | 11 | *Klebsiella* | *Klebsiella pneumoniae* subsp. pneumoniae KPNIH24, complete genome |
| CP008827 | 1 | 11 | *Klebsiella* | *Klebsiella pneumoniae* subsp. pneumoniae KPNIH1, complete genome |
| CP008831 | 1 | 11 | *Klebsiella* | *Klebsiella pneumoniae* subsp. pneumoniae KPR0928, complete sequence |
| CP008929 | 1 | 11 | *Klebsiella* | *Klebsiella pneumoniae* PMK1, complete genome |
| CP009114 | 1 | 11 | *Klebsiella* | *Klebsiella pneumoniae* blaNDM-1, complete genome |
| CP009208 | 1 | 11 | *Klebsiella* | *Klebsiella pneumoniae* subsp. pneumoniae ATCC 43816 KPPR1, complete genome |
| CP009461 | 1 | 11 | *Klebsiella* | *Klebsiella pneumoniae* XH209, complete genome |
| CP009771 | 1 | 11 | *Klebsiella* | *Klebsiella pneumoniae* subsp. pneumoniae KPNIH33, complete genome |
| CP009775 | 1 | 11 | *Klebsiella* | *Klebsiella pneumoniae* subsp. pneumoniae KPNIH32, complete genome |
| CP009863 | 1 | 11 | *Klebsiella* | *Klebsiella pneumoniae* subsp. pneumoniae KPNIH29, complete genome |
| CP009872 | 1 | 11 | *Klebsiella* | *Klebsiella pneumoniae* subsp. pneumoniae KPNIH30, complete genome |
| CP009876 | 1 | 11 | *Klebsiella* | *Klebsiella pneumoniae* subsp. pneumoniae KPNIH31, complete genome |
| CP010361 | 1 | 11 | *Klebsiella* | *Klebsiella pneumoniae* 32192, complete genome |
| CP010392 | 1 | 11 | *Klebsiella* | *Klebsiella pneumoniae* 34618, complete genome |
| CP010523 | 1 | 11 | *Klebsiella* | *Klebsiella variicola* DSM 15968, complete genome |
| CP011313 | 1 | 11 | *Klebsiella* | *Klebsiella pneumoniae* subsp. pneumoniae 234-12, complete genome |
| CP011421 | 1 | 11 | *Klebsiella* | *Klebsiella pneumoniae* yzusk-4 genome |
| CP011578 | 1 | 11 | *Klebsiella* | *Klebsiella pneumoniae* CAV1392, complete genome |
| CP011624 | 1 | 11 | *Klebsiella* | *Klebsiella pneumoniae* CAV1344, complete genome |
| CP011647 | 1 | 11 | *Klebsiella* | *Klebsiella pneumoniae* CAV1596, complete genome |
| CP011976 | 1 | 11 | *Klebsiella* | *Klebsiella pneumoniae* DMC1097, complete genome |
| CP011980 | 1 | 11 | *Klebsiella* | *Klebsiella pneumoniae* 500_1420, complete genome |
| CP011985 | 1 | 11 | *Klebsiella* | *Klebsiella pneumoniae* UHKPC07, complete genome |
| CP011989 | 1 | 11 | *Klebsiella* | *Klebsiella pneumoniae* UHKPC33, complete genome |
| CP012043 | 1 | 11 | *Klebsiella* | *Klebsiella pneumoniae* U25 genome |
| CP012252 | 1 | 11 | *Klebsiella* | *Klebsiella variicola* HKUOPLA, complete genome |
| CP012300 | 1 | 11 | *Klebsiella* | *Klebsiella pneumoniae* subsp. pneumoniae HKUOPLC, complete genome |
| CP012426 | 1 | 11 | *Klebsiella* | *Klebsiella pneumoniae* KP5, complete genome |
| CP012560 | 1 | 11 | *Klebsiella* | *Klebsiella pneumoniae* UCLAOXA232KP_Pt0, complete genome |
| CP012561 | 1 | 11 | *Klebsiella* | *Klebsiella pneumoniae* UCLAOXA232KP, complete genome |
| CP012568 | 1 | 11 | *Klebsiella* | *Klebsiella pneumoniae* UCLAOXA232KP, complete genome |
| CP012743 | 1 | 11 | *Klebsiella* | *Klebsiella pneumoniae* subsp. pneumoniae TGH8, complete genome |
| CP012744 | 1 | 11 | *Klebsiella* | *Klebsiella pneumoniae* subsp. pneumoniae TGH10, complete genome |
| CP012745 | 1 | 11 | *Klebsiella* | *Klebsiella pneumoniae* subsp. pneumoniae TGH13, complete genome |
| CP012753 | 1 | 11 | *Klebsiella* | *Klebsiella pneumoniae* KP617, complete genome |
| CP012883 | 1 | 11 | *Klebsiella* | *Klebsiella pneumoniae* KP-1, complete genome |
| CP012987 | 1 | 11 | *Klebsiella* | *Klebsiella pneumoniae* KpN01, complete genome |
| CP012992 | 1 | 11 | *Klebsiella* | *Klebsiella pneumoniae* KpN06, complete genome |
| CP013322 | 1 | 11 | *Klebsiella* | *Klebsiella pneumoniae* CAV1193, complete genome |
| CP013711 | 1 | 11 | *Klebsiella* | *Klebsiella pneumoniae* J1, complete genome |
| CP014004 | 1 | 11 | *Klebsiella* | *Klebsiella pneumoniae* subsp. pneumoniae NUHL24835, complete genome |
| CP014008 | 1 | 11 | *Klebsiella* | *Klebsiella pneumoniae* subsp. pneumoniae RJF293, complete genome |
| CP014010 | 1 | 11 | *Klebsiella* | *Klebsiella pneumoniae* subsp. pneumoniae RJF999, complete genome |
| CP014123 | 1 | 11 | *Klebsiella* | *Klebsiella pneumoniae* FDAARGOS_156, complete genome |
| CP014154 | 1 | 11 | *Klebsiella* | *Klebsiella quasipneumoniae* HKUOPA4, complete genome |
| CP014155 | 1 | 11 | *Klebsiella* | *Klebsiella quasipneumoniae* HKUOPJ4, complete genome |
| CP014156 | 1 | 11 | *Klebsiella* | *Klebsiella quasipneumoniae* HKUOPL4, complete genome |
| CP014294 | 1 | 11 | *Klebsiella* | *Klebsiella pneumoniae* KP38731, complete genome |
| CP014647 | 1 | 11 | *Klebsiella* | *Klebsiella pneumoniae* KPNIH36, complete genome |
| CP014755 | 1 | 11 | *Klebsiella* | *Klebsiella pneumoniae* AATZP, complete genome |
| CP014762 | 1 | 11 | *Klebsiella* | *Klebsiella pneumoniae* KPNIH39, complete genome |
| CP015025 | 1 | 11 | *Klebsiella* | *Klebsiella pneumoniae* Kpn223, complete genome |
| CP015120 | 1 | 11 | *Klebsiella* | *Klebsiella pneumoniae* kp757, complete genome |
| CP015130 | 1 | 11 | *Klebsiella* | *Klebsiella pneumoniae* Kpn555, complete genome |
| CP015134 | 1 | 11 | *Klebsiella* | *Klebsiella pneumoniae* ATCC 35657, complete genome |
| CP015382 | 1 | 11 | *Klebsiella* | *Klebsiella pneumoniae* CN1, complete genome |
| CP015385 | 1 | 11 | *Klebsiella* | *Klebsiella pneumoniae* NY9, complete genome |
| CP015392 | 1 | 11 | *Klebsiella* | *Klebsiella pneumoniae* CR14, complete genome |
| CP015500 | 1 | 11 | *Klebsiella* | *Klebsiella pneumoniae* SKGH01, complete genome |
| CP015753 | 1 | 11 | *Klebsiella* | *Klebsiella pneumoniae* W14, complete genome |
| CP015822 | 1 | 11 | *Klebsiella* | *Klebsiella pneumoniae* isolate blood sample 2, complete genome |
| CP015990 | 1 | 11 | *Klebsiella* | *Klebsiella pneumoniae* BR, complete genome |
| CP016159 | 1 | 11 | *Klebsiella* | *Klebsiella pneumoniae* TH1, complete genome |
| CP016811 | 1 | 11 | *Klebsiella* | *Klebsiella pneumoniae* DHQP1002001, complete genome |
| CP016813 | 1 | 11 | *Klebsiella* | *Klebsiella pneumoniae* ED2, complete genome |
| CP016814 | 1 | 11 | *Klebsiella* | *Klebsiella pneumoniae* ED23, complete genome |
| CP016923 | 1 | 11 | *Klebsiella* | *Klebsiella pneumoniae* isolate 11, complete genome |
| CP016926 | 1 | 11 | *Klebsiella* | *Klebsiella pneumoniae* isolate 23, complete genome |
| CP017284 | 1 | 11 | *Klebsiella* | *Klebsiella variicola* GJ1, complete genome |
| CP017289 | 1 | 11 | *Klebsiella* | *Klebsiella variicola* GJ3, complete genome |
| CP017385 | 1 | 11 | *Klebsiella* | *Klebsiella pneumoniae* KP36, complete genome |
| CP017849 | 1 | 11 | *Klebsiella* | *Klebsiella variicola* GJ2, complete genome |
| CP017934 | 1 | 11 | *Klebsiella* | *Klebsiella pneumoniae* CAV1016, complete genome |
| CP017985 | 1 | 11 | *Klebsiella* | *Klebsiella pneumoniae* 825795-1, complete genome |
| CP017994 | 1 | 11 | *Klebsiella* | *Klebsiella pneumoniae* P1428, complete genome |
| CP018056 | 1 | 11 | *Klebsiella* | *Klebsiella pneumoniae* subsp. pneumoniae H11, complete genome |
| CP018140 | 1 | 11 | *Klebsiella* | *Klebsiella pneumoniae* Kp_Goe_822579, complete genome |
| CP018306 | 1 | 11 | *Klebsiella* | *Klebsiella pneumoniae* 459, complete genome |
| CP018337 | 1 | 11 | *Klebsiella* | *Klebsiella pneumoniae* Kp_Goe_154414, complete genome |
| CP018352 | 1 | 11 | *Klebsiella* | *Klebsiella pneumoniae* CAV1417, complete genome |
| CP018356 | 1 | 11 | *Klebsiella* | *Klebsiella pneumoniae* CAV1453, complete genome |
| CP018364 | 1 | 11 | *Klebsiella* | *Klebsiella pneumoniae* Kp_Goe_62629, complete genome |
| CP018427 | 1 | 11 | *Klebsiella* | *Klebsiella pneumoniae* MNCRE69, complete genome |
| CP018428 | 1 | 11 | *Klebsiella* | *Klebsiella pneumoniae* MNCRE78, complete genome |
| CP018437 | 1 | 11 | *Klebsiella* | *Klebsiella pneumoniae* MNCRE53, complete genome |
| CP018438 | 1 | 11 | *Klebsiella* | *Klebsiella pneumoniae* Kp_Goe_822917, complete sequence |
| CP018447 | 1 | 11 | *Klebsiella* | *Klebsiella pneumoniae* Kp_Goe_33208, complete genome |
| CP018450 | 1 | 11 | *Klebsiella* | *Klebsiella pneumoniae* Kp_Goe_71070, complete genome |
| CP018454 | 1 | 11 | *Klebsiella* | *Klebsiella pneumoniae* SWU01, complete genome |
| CP018458 | 1 | 11 | *Klebsiella* | *Klebsiella pneumoniae* Kp_Goe_39795, complete genome |
| CP018671 | 1 | 11 | *Klebsiella* | *Klebsiella pneumoniae* CAV1042, complete genome |
| CP018676 | 1 | 11 | *Klebsiella* | *Klebsiella pneumoniae* CAV1217, complete genome |
| CP018686 | 1 | 11 | *Klebsiella* | *Klebsiella pneumoniae* Kp_Goe_149473, complete sequence |
| CP018692 | 1 | 11 | *Klebsiella* | *Klebsiella pneumoniae* Kp_Goe_821588, complete sequence |
| CP018695 | 1 | 11 | *Klebsiella* | *Klebsiella pneumoniae* Kp_Goe_149832, complete genome |
| CP018701 | 1 | 11 | *Klebsiella* | *Klebsiella pneumoniae* Kp_Goe_827024, complete genome |
| CP018707 | 1 | 11 | *Klebsiella* | *Klebsiella pneumoniae* Kp_Goe_827026, complete genome |
| CP018713 | 1 | 11 | *Klebsiella* | *Klebsiella pneumoniae* Kp_Goe_152021, complete genome |
| CP018719 | 1 | 11 | *Klebsiella* | *Klebsiella pneumoniae* KP_Goe_828304, complete genome |
| CP018735 | 1 | 11 | *Klebsiella* | *Klebsiella pneumoniae* Kp_Goe_121641, complete genome |
| CP018816 | 1 | 11 | *Klebsiella* | *Klebsiella pneumoniae* AR_0049, complete genome |
| CP018883 | 1 | 11 | *Klebsiella* | *Klebsiella pneumoniae* subsp. pneumoniae BR7, complete genome |
| CP018885 | 1 | 11 | *Klebsiella* | *Klebsiella pneumoniae* subsp. pneumoniae BR21, complete genome |
| CP019047 | 1 | 11 | *Klebsiella* | *Klebsiella pneumoniae* subsp. pneumoniae RJA166, complete genome |
| CP019077 | 1 | 11 | *Klebsiella* | *Klebsiella pneumoniae* DT1 |
| CP019079 | 1 | 11 | *Klebsiella* | *Klebsiella pneumoniae* DT12 |
| CP019160 | 1 | 11 | *Klebsiella* | *Klebsiella pneumoniae* GN-2, complete genome |
| CP019219 | 1 | 11 | *Klebsiella* | *Klebsiella pneumoniae* 1756, complete genome |
| CP019772 | 1 | 11 | *Klebsiella* | *Klebsiella pneumoniae* subsp. pneumoniae KPN_KPC_HUG_07 genome |
| CP020061 | 1 | 11 | *Klebsiella* | *Klebsiella pneumoniae* AR_0117, complete genome |
| CP020067 | 1 | 11 | *Klebsiella* | *Klebsiella pneumoniae* AR_0068, complete genome |
| CP020071 | 1 | 11 | *Klebsiella* | *Klebsiella pneumoniae* AR_0115, complete genome |
| CP020108 | 1 | 11 | *Klebsiella* | *Klebsiella pneumoniae* AR_0098, complete genome |
| CP020837 | 1 | 11 | *Klebsiella* | *Klebsiella pneumoniae* BK13043, complete genome |
| CP020841 | 1 | 11 | *Klebsiella* | *Klebsiella pneumoniae* KPN1482, complete genome |
| CP020847 | 1 | 11 | *Klebsiella* | *Klebsiella pneumoniae* KPN1481, complete genome |
| CP020853 | 1 | 11 | *Klebsiella* | *Klebsiella pneumoniae* KPN528, complete genome |
| CP020901 | 1 | 11 | *Klebsiella* | *Klebsiella pneumoniae* K66-45, complete genome |
| CP021539 | 1 | 11 | *Klebsiella* | *Klebsiella pneumoniae* AR_0047, complete genome |
| CP021549 | 1 | 11 | *Klebsiella* | *Klebsiella pneumoniae* AR_0112, complete genome |
| CP021685 | 1 | 11 | *Klebsiella* | *Klebsiella pneumoniae* AR_0146, complete genome |
| CP021696 | 1 | 11 | *Klebsiella* | *Klebsiella pneumoniae* AR_0158, complete genome |
| CP021708 | 1 | 11 | *Klebsiella* | *Klebsiella pneumoniae* AR_0143, complete genome |
| CP021718 | 1 | 11 | *Klebsiella* | *Klebsiella pneumoniae* AR_0129, complete genome |
| CP021740 | 1 | 11 | *Klebsiella* | *Klebsiella pneumoniae* AR_0126, complete genome |
| CP021751 | 1 | 11 | *Klebsiella* | *Klebsiella pneumoniae* AR_0113, complete genome |
| CP021757 | 1 | 11 | *Klebsiella* | *Klebsiella pneumoniae* AR_0138, complete genome |
| CP021833 | 1 | 11 | *Klebsiella* | *Klebsiella pneumoniae* AR_0120, complete genome |
| CP021859 | 1 | 11 | *Klebsiella* | *Klebsiella pneumoniae* AR_0125, complete genome |
| CP021939 | 1 | 11 | *Klebsiella* | *Klebsiella pneumoniae* AR_0145, complete genome |
| CP021944 | 1 | 11 | *Klebsiella* | *Klebsiella pneumoniae* AR_0152, complete genome |
| CP021950 | 1 | 11 | *Klebsiella* | *Klebsiella pneumoniae* AR_0148, complete genome |
| CP021955 | 1 | 11 | *Klebsiella* | *Klebsiella pneumoniae* AR_0107, complete genome |
| CP021960 | 1 | 11 | *Klebsiella* | *Klebsiella pneumoniae* AR_0139, complete genome |
| CP022023 | 1 | 11 | *Klebsiella* | *Klebsiella pneumoniae* 19051, complete genome |
| CP022127 | 1 | 11 | *Klebsiella* | *Klebsiella pneumoniae* DHQP1605752_NV, complete genome |
| CP022143 | 1 | 11 | *Klebsiella* | *Klebsiella pneumoniae* 704SK6 genome |
| CP022573 | 1 | 11 | *Klebsiella* | *Klebsiella pneumoniae* BIC-1, complete genome |
| CP022691 | 1 | 11 | *Klebsiella* | *Klebsiella pneumoniae* subsp. pneumoniae AUSMDU00008079, complete genome |
| CP022823 | 1 | 11 | *Klebsiella* | Klebsiella quasivariicola KPN1705, complete genome |
| CP022882 | 1 | 11 | *Klebsiella* | *Klebsiella pneumoniae* 911021, complete genome |
| CP022997 | 1 | 11 | *Klebsiella* | *Klebsiella pneumoniae* 721005, complete genome |
| CP023134 | 1 | 11 | *Klebsiella* | *Klebsiella pneumoniae* subsp. pneumoniae KpvK54, complete genome |
| CP023487 | 1 | 11 | *Klebsiella* | *Klebsiella pneumoniae* subsp. pneumoniae ST101:960186733, complete genome |
| CP023502 | 1 | 11 | *Klebsiella* | *Klebsiella pneumoniae* FDAARGOS_445, complete genome |
| CP023553 | 1 | 11 | *Klebsiella* | *Klebsiella pneumoniae* subsp. pneumoniae ST2017:950142398 |
| CP023907 | 1 | 11 | *Klebsiella* | *Klebsiella pneumoniae* FDAARGOS_436, complete genome |
| CP023913 | 1 | 11 | *Klebsiella* | *Klebsiella pneumoniae* FDAARGOS_439, complete genome |
| CP023919 | 1 | 11 | *Klebsiella* | *Klebsiella pneumoniae* FDAARGOS_440, complete genome |
| CP023925 | 1 | 11 | *Klebsiella* | *Klebsiella pneumoniae* FDAARGOS_442, complete genome |
| CP023933 | 1 | 11 | *Klebsiella* | *Klebsiella pneumoniae* FDAARGOS_443, complete genome |
| CP023941 | 1 | 11 | *Klebsiella* | *Klebsiella pneumoniae* FDAARGOS_444, complete genome |
| CP023946 | 1 | 11 | *Klebsiella* | *Klebsiella pneumoniae* FDAARGOS_446, complete genome |
| CP023949 | 1 | 11 | *Klebsiella* | *Klebsiella pneumoniae* FDAARGOS_447, complete genome |
| CP024038 | 1 | 11 | *Klebsiella* | *Klebsiella pneumoniae* QS17-0029, complete genome |
| CP024191 | 1 | 11 | *Klebsiella* | *Klebsiella pneumoniae* KSB1_5D, complete genome |
| CP024458 | 1 | 11 | *Klebsiella* | *Klebsiella pneumoniae* QS17-0161, complete genome |
| CP024482 | 1 | 11 | *Klebsiella* | *Klebsiella pneumoniae* INF322, complete genome |
| CP024489 | 1 | 11 | *Klebsiella* | *Klebsiella pneumoniae* INF249, complete genome |
| CP024496 | 1 | 11 | *Klebsiella* | *Klebsiella pneumoniae* KSB1_7E, complete genome |
| CP024499 | 1 | 11 | *Klebsiella* | *Klebsiella pneumoniae* KSB1_4E, complete genome |
| CP024504 | 1 | 11 | *Klebsiella* | *Klebsiella pneumoniae* KSB2_1B, complete genome |
| CP024515 | 1 | 11 | *Klebsiella* | *Klebsiella pneumoniae* KSB1_10J, complete genome |
| CP024521 | 1 | 11 | *Klebsiella* | *Klebsiella pneumoniae* INF158, complete genome |
| CP024528 | 1 | 11 | *Klebsiella* | *Klebsiella pneumoniae* INF157, complete genome |
| CP024535 | 1 | 11 | *Klebsiella* | *Klebsiella pneumoniae* KSB1_9D, complete genome |
| CP024542 | 1 | 11 | *Klebsiella* | *Klebsiella pneumoniae* INF042, complete genome |
| CP024545 | 1 | 11 | *Klebsiella* | *Klebsiella pneumoniae* INF059, complete genome |
| CP024548 | 1 | 11 | *Klebsiella* | *Klebsiella pneumoniae* KSB1_7J, complete genome |
| CP024549 | 1 | 11 | *Klebsiella* | *Klebsiella pneumoniae* INF163, complete genome |
| CP024556 | 1 | 11 | *Klebsiella* | *Klebsiella pneumoniae* INF164, complete genome |
| CP024563 | 1 | 11 | *Klebsiella* | *Klebsiella pneumoniae* INF278, complete genome |
| CP024570 | 1 | 11 | *Klebsiella* | *Klebsiella pneumoniae* INF274, complete genome |
| CP024834 | 1 | 11 | *Klebsiella* | *Klebsiella pneumoniae* CRKP-2297, complete genome |
| CP024838 | 1 | 11 | *Klebsiella* | *Klebsiella pneumoniae* CRKP-1215, complete genome |
| CP025005 | 1 | 11 | *Klebsiella* | *Klebsiella pneumoniae* AUSMDU00003562, complete genome |
| CP025008 | 1 | 11 | *Klebsiella* | *Klebsiella pneumoniae* AUSMDU00008119, complete genome |
| CP025037 | 1 | 11 | *Klebsiella* | *Klebsiella pneumoniae* NU-CRE047, complete genome |
| CP025080 | 1 | 11 | *Klebsiella* | *Klebsiella pneumoniae* SGH10, complete genome |
| CP025087 | 1 | 11 | *Klebsiella* | *Klebsiella pneumoniae* KP6 |
| CP025088 | 1 | 11 | *Klebsiella* | *Klebsiella pneumoniae* KP7 |
| CP025089 | 1 | 11 | *Klebsiella* | *Klebsiella pneumoniae* KP8 |
| CP025090 | 1 | 11 | *Klebsiella* | *Klebsiella pneumoniae* KP9 |
| CP025091 | 1 | 11 | *Klebsiella* | *Klebsiella pneumoniae* KP10 |
| CP025092 | 1 | 11 | *Klebsiella* | *Klebsiella pneumoniae* KP11 |
| CP025093 | 1 | 11 | *Klebsiella* | *Klebsiella pneumoniae* KP14 |
| CP025140 | 1 | 11 | *Klebsiella* | *Klebsiella pneumoniae* KP1768, complete genome |
| CP025143 | 1 | 11 | *Klebsiella* | *Klebsiella pneumoniae* NR5632, complete genome |
| CP025146 | 1 | 11 | *Klebsiella* | *Klebsiella pneumoniae* KP1766, complete genome |
| CP025211 | 1 | 11 | *Klebsiella* | *Klebsiella pneumoniae* HZW25, complete genome |
| CP025456 | 1 | 11 | *Klebsiella* | *Klebsiella pneumoniae* KP69, complete genome |
| CP025461 | 1 | 11 | *Klebsiella* | *Klebsiella pneumoniae* F44, complete genome |
| CP025466 | 1 | 11 | *Klebsiella* | *Klebsiella pneumoniae* JS187, complete genome |
| CP025515 | 1 | 11 | *Klebsiella* | *Klebsiella pneumoniae* 002SK2, complete genome |
| CP025541 | 1 | 11 | *Klebsiella* | *Klebsiella sp*. 2N3, complete genome |
| CP025629 | 1 | 11 | *Klebsiella* | *Klebsiella pneumoniae* LS358 |
| CP025630 | 1 | 11 | *Klebsiella* | *Klebsiella pneumoniae* LS359 |
| CP025631 | 1 | 11 | *Klebsiella* | *Klebsiella pneumoniae* HS09565 |
| CP025633 | 1 | 11 | *Klebsiella* | *Klebsiella pneumoniae* HS102438 |
| CP025639 | 1 | 11 | *Klebsiella* | *Klebsiella pneumoniae* LS357 |
| CP025641 | 1 | 11 | *Klebsiella* | *Klebsiella pneumoniae* LS355 |
| CP025816 | 1 | 11 | *Klebsiella* | *Klebsiella pneumoniae* Kp81 |
| CP025951 | 1 | 11 | *Klebsiella* | *Klebsiella pneumoniae* subsp. pneumoniae GD4, complete genome |
| CP025963 | 1 | 11 | *Klebsiella* | *Klebsiella pneumoniae* WCHKP34, complete genome |
| CP026177 | 1 | 11 | *Klebsiella* | *Klebsiella pneumoniae* KPNIH50, complete genome |
| CP026178 | 1 | 11 | *Klebsiella* | *Klebsiella pneumoniae* KPNIH49, complete genome |
| CP026392 | 1 | 11 | *Klebsiella* | *Klebsiella pneumoniae* KPNIH48, complete genome |
| CP026585 | 1 | 11 | *Klebsiella* | *Klebsiella pneumoniae* WCHKP649, complete genome |
| CP026586 | 1 | 11 | *Klebsiella* | *Klebsiella pneumoniae* NUHL30457, complete genome |
| CP026751 | 1 | 11 | *Klebsiella* | *Klebsiella pneumoniae* AR_0066, complete genome |
| CP027064 | 1 | 11 | *Klebsiella* | *Klebsiella variicola* WCHKV030666, complete genome |
| CP027068 | 1 | 11 | *Klebsiella* | *Klebsiella pneumoniae* WCHKP8F4, complete genome |
| CP027146 | 1 | 11 | *Klebsiella* | *Klebsiella pneumoniae* AR_0363, complete genome |
| CP027160 | 1 | 11 | *Klebsiella* | *Klebsiella pneumoniae* AR_0361, complete genome |
| CP027189 | 1 | 11 | *Klebsiella* | *Klebsiella pneumoniae* KPHS1249, complete genome |
| FO203501 | 1 | 11 | *Klebsiella* | *Klebsiella pneumoniae* subsp. rhinoscleromatis SB3432, complete genome |
| FO834906 | 1 | 11 | *Klebsiella* | *Klebsiella pneumoniae* str. Kp52.145,, complete genome |
| LN824133 | 1 | 11 | *Klebsiella* | *Klebsiella pneumoniae* genome assembly MS6671.v1, : _Chr_Kpneumoniae_MS6671 |
| LT216436 | 1 | 11 | *Klebsiella* | *Klebsiella pneumoniae* isolate 207M1D0-sc-2013-04-03T11:21:06Z-1606409 genome assembly,: 1 |
| r46mdfa | 1 | 11^a^ | *Klebsiella* | this study |
| CP009274 | 2 | 11 | *Klebsiella* | *Klebsiella variicola* DX120E, complete genome |
| CP014071 | 2 | 11 | *Klebsiella* | *Klebsiella quasipneumoniae* ATCC 700603, complete genome |
| CP014696 | 2 | 11 | *Klebsiella* | *Klebsiella quasipneumoniae* ATCC 700603, complete genome |
| CP008700 | 3 | 11 | *Klebsiella* | *Klebsiella pneumoniae* subsp. pneumoniae KP5-1, complete genome |
| CP013985 | 3 | 11 | *Klebsiella* | *Klebsiella variicola* LMG 23571, complete genome |
| CP020657 | 4 | 11 | *Klebsiella* | *Klebsiella sp*. M5al, complete genome |
| CP008788 | 5 | 11 | *Klebsiella* | *Klebsiella oxytoca* KONIH1, complete genome |
| CP008841 | 5 | 11 | *Klebsiella* | *Klebsiella michiganensis* M1, complete genome |
| CP026269 | 5 | 11 | *Klebsiella* | *Klebsiella oxytoca* KONIH4, complete genome |
| CP003683 | 6 | 11 | *Klebsiella* | *Klebsiella michiganensis* E718, complete genome |
| CP011597 | 6 | 11 | *Klebsiella* | *Klebsiella oxytoca* CAV1099, complete genome |
| CP011618 | 6 | 11 | *Klebsiella* | *Klebsiella oxytoca* CAV1335, complete genome |
| CP011636 | 6 | 11 | *Klebsiella* | *Klebsiella oxytoca* CAV1374, complete genome |
| CP017450 | 6 | 11 | *Klebsiella* | *Klebsiella sp*. LTGPAF-6F, complete genome |
| CP017928 | 6 | 11 | *Klebsiella* | *Klebsiella oxytoca* CAV1015, complete genome |
| CP018362 | 6 | 11 | *Klebsiella* | *Klebsiella oxytoca* CAV1752, complete genome |
| CP020358 | 6 | 11 | *Klebsiella* | *Klebsiella oxytoca* AR_0147, complete genome |
| CP022348 | 6 | 11 | *Klebsiella* | *Klebsiella michiganensis* K516, complete genome |
| CP023185 | 6 | 11 | *Klebsiella* | *Klebsiella michiganensis* K518, complete genome |
| CP026275 | 6 | 11 | *Klebsiella* | *Klebsiella oxytoca* KONIH5, complete genome |
| CP026285 | 6 | 11 | *Klebsiella* | *Klebsiella oxytoca* KONIH2, complete genome |
| CP026715 | 6 | 11 | *Klebsiella* | *Klebsiella oxytoca* AR_0028, complete genome |
| CP003218 | 7 | 11 | *Klebsiella* | *Klebsiella michiganensis* KCTC 1686, complete genome |
| CP004887 | 7 | 11 | *Klebsiella* | *Klebsiella michiganensis* HKOPL1, complete genome |
| CP010557 | 8 | 11 | *Raoultella* | *Raoultella ornithinolytica* S12, complete genome |
| CP023525 | 9 | 4 | *Cedecea* | *Cedecea neteri* FDAARGOS_392, complete genome |
| CP002272 | 10 | 11 | *Pluralibacter* | *Enterobacter lignolyticus* SCF1, complete genome |
| CP012871 | 10 | 11 | *Pluralibacter* | [*Enterobacter*] *lignolyticus* G5, complete genome |
| CP004142 | 11 | 3 | *Raoultella* | *Raoultella ornithinolytica* B6, complete genome |
| CP008886 | 11 | 3 | *Raoultella* | *Raoultella ornithinolytica* A14, complete genome |
| CP012555 | 11 | 3 | *Raoultella* | *Raoultella ornithinolytica* 18 sequence |
| CP013338 | 11 | 3 | *Raoultella* | *Raoultella ornithinolytica* Yangling I2, complete genome |
| CP017802 | 11 | 3 | *Raoultella* | *Raoultella ornithinolytica* MG, complete genome |
| CP019899 | 11 | 3 | *Raoultella* | *Raoultella planticola* GODA, complete genome |
| CP021329 | 11 | 3 | *Raoultella* | *Raoultella ornithinolytica* Ro24724, complete genome |
| CP023874 | 11 | 3 | *Raoultella* | *Raoultella planticola* FDAARGOS_429, complete genome |
| CP023877 | 11 | 3 | *Raoultella* | *Raoultella planticola* FDAARGOS_430, complete genome |
| CP023888 | 11 | 3 | *Raoultella* | *Raoultella ornithinolytica* FDAARGOS_431, complete genome |
| CP026047 | 11 | 3 | *Raoultella* | *Raoultella planticola* FDAARGOS_64, complete genome |
| CP002824 | 12 | 3 | *klebsiella* | *Enterobacter aerogenes* KCTC 2190, complete genome |
| CP011539 | 12 | 3 | *Klebsiella* | *Klebsiella aerogenes* G7, complete genome |
| CP011574 | 12 | 3^a^ | *Klebsiella* | *Klebsiella aerogenes* CAV1320, complete genome |
| CP014029 | 12 | 3 | *Klebsiella* | *Klebsiella aerogenes* FDAARGOS_152, complete genome |
| CP014748 | 12 | 3 | *Klebsiella* | *Klebsiella aerogenes* FDAARGOS_139, complete genome |
| CP023963 | 12 | 3 | *Klebsiella* | *Klebsiella aerogenes* FDAARGOS_363, complete genome |
| CP024880 | 12 | 3 | *Klebsiella* | *Klebsiella aerogenes* AR_0018, complete genome |
| CP024883 | 12 | 3 | *Klebsiella* | *Klebsiella aerogenes* AR_0007, complete genome |
| CP024885 | 12 | 3 | *Klebsiella* | *Klebsiella aerogenes* AR_0009, complete genome |
| CP026756 | 12 | 3 | *Klebsiella* | *Klebsiella aerogenes* AR_0062, complete genome |
| FO203355 | 12 | 3 | *Klebsiella* | *Enterobacter aerogenes* EA1509E complete genome |
| CP011077 | 13 | 4 | *Klebsiella* | *Klebsiella michiganensis* RC10, complete genome |
| CP026193 | 14 | 8^a^ | unknow^b^ | Enterobacteriaceae bacterium ENNIH1, complete genome |
| CP009458 | 15 | 5 | *Cedecea* | *Cedecea neteri* M006, complete genome |
| CP026192 | 16 | 6^a^ | unknow^b^ | Enterobacteriaceae bacterium ENNIH2, complete genome |
| CP026197 | 16 | 6 | unknow^b^ | Enterobacteriaceae bacterium ENNIH3, complete genome |
| CP009459 | 17 | 5^a^ | *Cedecea* | *Cedecea neteri* ND14a, complete genome |
| CP012266 | 18 | 1 | *Cronobacter* | *Cronobacter dublinensis* subsp. dublinensis LMG 23823, complete genome |
| CP012268 | 18 | 1 | *Cronobacter* | Cronobacter muytjensii ATCC 51329, complete genome |
| CP009451 | 19 | 4^a^ | *Cedecea* | *Cedecea neteri* SSMD04, complete genome |
| CP019445 | 20 | 9 | *Kosakonia* | *Kosakonia cowanii* JCM 10956 = DSM 18146 888-76, complete genome |
| CP022690 | 20 | 9 | *Kosakonia* | *Kosakonia cowanii* Esp_Z genome |
| CP013990 | 21 | 2 | *Leclercia* | *Leclercia adecarboxylata* USDA-ARS-USMARC-60222, complete genome |
| CP026387 | 21 | 2 | *Leclercia* | Leclercia sp. LSNIH3, complete genome |
| CP003938 | 22 | 11 | unknow^b^ | Enterobacteriaceae bacterium FGI 57, complete genome |
| CP021851 | 23 | 2 | *Enterobacter cloacae complex* | *Enterobacter cloacae* A1137, complete genome |
| CP026167 | 24 | 2 | *Leclercia* | Leclercia sp. LSNIH1, complete genome |
| CP002886 | 25 | 2 | *Enterobacter cloacae complex* | *Enterobacter cloacae* EcWSU1, complete genome |
| CP006580 | 25 | 2 | *Enterobacter cloacae complex* | *Enterobacter cloacae* P101, complete genome |
| CP011798 | 25 | 2 | *Enterobacter cloacae complex* | *Enterobacter cloacae* UW5, complete genome |
| CP017279 | 25 | 2 | *Enterobacter cloacae complex* | *Enterobacter ludwigii* EN-119, complete genome |
| CP018785 | 25 | 2 | *Enterobacter cloacae complex* | *Enterobacter cloacae* AA4, complete genome |
| CP011602 | 26 | 8 | *Kluyvera* | Kluyvera intermedia CAV1151, complete genome |
| CP025034 | 27 | 2 | *Lelliottia* | Lelliottia nimipressuralis SGAir0187, complete genome |
| CP010512 | 28 | 2 | *Enterobacter cloacae complex* | *Enterobacter cloacae* colR/S, complete genome |
| CP011591 | 28 | 2 | *Enterobacter cloacae complex* | *Enterobacter asburiae* CAV1043, complete genome |
| CP014993 | 28 | 2 | *Enterobacter cloacae complex* | *Enterobacter asburiae* ENIPBJ-CG1, complete genome |
| CP017990 | 28 | 2 | *Enterobacter cloacae complex* | *Enterobacter cloacae* complex sp. ECNIH7, complete genome |
| CP026975 | 28 | 2^a^ | *Enterobacter cloacae complex* | *Enterobacter cloacae* complex bacterium FDAARGOS_77, complete genome |
| CP012162 | 29 | 2 | *Enterobacter cloacae complex* | *Enterobacter cloacae* complex sp. 35734 1, complete sequence |
| CP017184 | 29 | 2 | *Enterobacter cloacae complex* | *Enterobacter cloacae* complex 'Hoffmann cluster IV' DSM 16690, complete genome |
| CP019839 | 29 | 2 | *Enterobacter cloacae complex* | *Enterobacter cloacae* R11, complete genome |
| CP022148 | 29 | 2 | *Enterobacter cloacae complex* | *Enterobacter cloacae* 704SK10, complete genome |
| CP007546 | 30 | 2 | *Enterobacter cloacae complex* | *Enterobacter asburiae* L1, complete genome |
| CP011863 | 30 | 2 | *Enterobacter cloacae complex* | *Enterobacter asburiae* ATCC 35953, complete sequence |
| CP001918 | 31 | 2 | *Enterobacter cloacae complex* | *Enterobacter cloacae* subsp. cloacae ATCC 13047, complete genome |
| CP003678 | 31 | 2 | *Enterobacter cloacae complex* | *Enterobacter cloacae* subsp. dissolvens SDM, complete genome |
| CP009756 | 31 | 2 | *Enterobacter cloacae complex* | *Enterobacter cloacae* GGT036, complete genome |
| CP016906 | 31 | 2 | *Enterobacter cloacae complex* | *Enterobacter cloacae* isolate SBP-8 genome |
| CP017475 | 31 | 2 | *Enterobacter cloacae complex* | *Enterobacter cloacae* M12X01451 complete genome |
| CP003737 | 32 | 2 | *Enterobacter cloacae complex* | *Enterobacter cloacae* subsp. cloacae ENHKU01, complete genome |
| CP009850 | 32 | 2 | *Enterobacter cloacae complex* | *Enterobacter cloacae* ECNIH4, complete genome |
| CP015227 | 32 | 2 | *Enterobacter cloacae complex* | *Enterobacter sp*. ODB01, complete genome |
| CP017181 | 32 | 2 | *Enterobacter cloacae complex* | Enterobacter kobei DSM 13645, complete genome |
| CP017087 | 33 | 2 | *Enterobacter cloacae complex* | *Enterobacter sp*. HK169, complete genome |
| CP020817 | 33 | 2 | *Enterobacter cloacae complex* | *Enterobacter sp*. Crenshaw, complete genome |
| CP012999 | 34 | 2 | *Enterobacter cloacae complex* | *Enterobacter sp*. E20, complete genome |
| CP023504 | 35 | 7^a^ | *Citrobacter* | *Citrobacter werkmanii* FDAARGOS_364, complete genome |
| CP014030 | 36 | 7 | *Citrobacter* | *Citrobacter sp*. FDAARGOS_156 FDAARGOS_155, complete genome |
| CP009450 | 37 | 11 | *Pluralibacter* | *Pluralibacter gergoviae* FB2, complete genome |
| CP012264 | 38 | 1 | *Cronobacter* | *Cronobacter condimenti* 1330 LMG 26250, complete genome |
| CP019113 | 39 | 9^a^ | *Enterobacter cloacae complex* | *Enterobacter sp*. SA187, complete genome |
| CP022695 | 40 | 11 | *Citrobacter* | *Citrobacter farmeri* AUSMDU00008141, complete genome |
| AE014075 | 41 | 7 | *Escherichia* | *Escherichia coli* CFT073, complete genome |
| AM946981 | 41 | 7 | *Escherichia* | *Escherichia coli* BL21(DE3), complete genome |
| AP009048 | 41 | 7 | *Escherichia* | *Escherichia coli* str. K-12 substr. W3110 DNA, complete genome |
| AP009378 | 41 | 7 | *Escherichia* | *Escherichia coli* SE15 DNA, complete genome |
| AP012030 | 41 | 7 | *Escherichia* | *Escherichia coli* DH1 (ME8569) DNA, complete genome |
| AP012306 | 41 | 7 | *Escherichia* | *Escherichia coli* str. K-12 substr. MDS42 DNA, complete genome |
| AP017610 | 41 | 7 | *Escherichia* | *Escherichia coli* DNA, complete genome, strain: 20Ec-P-124 |
| AP017617 | 41 | 7 | *Escherichia* | *Escherichia coli* DNA, complete genome, strain: MRY15-117 |
| AP017620 | 41 | 7 | *Escherichia* | *Escherichia coli* DNA, complete genome, strain: MRY15-131 |
| CP000243 | 41 | 7 | *Escherichia* | *Escherichia coli* UTI89, complete genome |
| CP000247 | 41 | 7 | *Escherichia* | *Escherichia coli* 536, complete genome |
| CP000468 | 41 | 7 | *Escherichia* | *Escherichia coli* APEC O1, complete genome |
| CP000802 | 41 | 7 | *Escherichia* | *Escherichia coli* HS, complete genome |
| CP000819 | 41 | 7 | *Escherichia* | *Escherichia coli* B str. REL606, complete genome |
| CP000946 | 41 | 7 | *Escherichia* | *Escherichia coli* ATCC 8739, complete genome |
| CP000948 | 41 | 7 | *Escherichia* | *Escherichia coli* str. K12 substr. DH10B, complete genome |
| CP000970 | 41 | 7 | *Escherichia* | *Escherichia coli* SMS-3-5, complete genome |
| CP001396 | 41 | 7 | *Escherichia* | *Escherichia coli* BW2952, complete genome |
| CP001509 | 41 | 7 | *Escherichia* | *Escherichia coli* BL21(DE3), complete genome |
| CP001637 | 41 | 7 | *Escherichia* | *Escherichia coli* DH1, complete genome |
| CP001665 | 41 | 7 | *Escherichia* | *Escherichia coli* 'BL21-Gold(DE3)pLysS AG', complete genome |
| CP001671 | 41 | 7 | *Escherichia* | *Escherichia coli* ABU 83972, complete genome |
| CP001855 | 41 | 7 | *Escherichia* | *Escherichia coli* O83:H1 str. NRG 857C, complete genome |
| CP001969 | 41 | 7 | *Escherichia* | *Escherichia coli* IHE3034, complete genome |
| CP002167 | 41 | 7 | *Escherichia* | *Escherichia coli* UM146, complete genome |
| CP002211 | 41 | 7 | *Escherichia* | *Escherichia coli* str. 'clone D i2', complete genome |
| CP002212 | 41 | 7 | *Escherichia* | *Escherichia coli* str. 'clone D i14', complete genome |
| CP002291 | 41 | 7 | *Escherichia* | *Escherichia coli* P12b, complete genome |
| CP002729 | 41 | 7 | *Escherichia* | *Escherichia coli* UMNK88, complete genome |
| CP005930 | 41 | 7 | *Escherichia* | *Escherichia coli* APEC IMT5155, complete genome |
| CP006636 | 41 | 7 | *Escherichia* | *Escherichia coli* PCN061, complete genome |
| CP006698 | 41 | 7 | *Escherichia* | Synthetic *Escherichia coli* C321.deltaA, complete sequence |
| CP006784 | 41 | 7 | *Escherichia* | *Escherichia coli* JJ1886, complete genome |
| CP006830 | 41 | 7 | *Escherichia* | *Escherichia coli* APEC O18, complete genome |
| CP006834 | 41 | 7 | *Escherichia* | *Escherichia coli* APEC O2-211, complete genome |
| CP007265 | 41 | 7 | *Escherichia* | *Escherichia coli* ST540, complete genome |
| CP007275 | 41 | 7 | *Escherichia* | *Escherichia coli* NMEC O18, complete genome |
| CP007390 | 41 | 7 | *Escherichia* | *Escherichia coli* ST540, complete genome |
| CP007391 | 41 | 7 | *Escherichia* | *Escherichia coli* ST540, complete genome |
| CP007799 | 41 | 7 | *Escherichia* | *Escherichia coli* Nissle 1917, complete genome |
| CP008697 | 41 | 7 | *Escherichia* | *Escherichia coli* ST648, complete genome |
| CP008801 | 41 | 7 | *Escherichia* | *Escherichia coli* KLY, complete genome |
| CP009072 | 41 | 7 | *Escherichia* | *Escherichia coli* ATCC 25922, complete genome |
| CP009166 | 41 | 7 | *Escherichia* | *Escherichia coli* 1303, complete genome |
| CP009273 | 41 | 7 | *Escherichia* | *Escherichia coli* BW25113, complete genome |
| CP009644 | 41 | 7 | *Escherichia* | *Escherichia coli* ER2796, complete genome |
| CP009685 | 41 | 7 | *Escherichia* | *Escherichia coli* str. K-12 substr. MG1655, complete genome |
| CP009789 | 41 | 7 | *Escherichia* | *Escherichia coli* K-12 ER3413, complete genome |
| CP009859 | 41 | 7 | *Escherichia* | *Escherichia coli* ECONIH1, complete genome |
| CP010116 | 41 | 7 | *Escherichia* | *Escherichia coli* C1, complete genome |
| CP010129 | 41 | 7 | *Escherichia* | *Escherichia coli* C9, complete genome |
| CP010137 | 41 | 7 | *Escherichia* | *Escherichia coli* D2, complete genome |
| CP010143 | 41 | 7 | *Escherichia* | *Escherichia coli* D4, complete genome |
| CP010151 | 41 | 7 | *Escherichia* | *Escherichia coli* D8, complete genome |
| CP010152 | 41 | 7 | *Escherichia* | *Escherichia coli* D9, complete genome |
| CP010157 | 41 | 7 | *Escherichia* | *Escherichia coli* D10, complete genome |
| CP010163 | 41 | 7 | *Escherichia* | *Escherichia coli* H2, complete genome |
| CP010169 | 41 | 7 | *Escherichia* | *Escherichia coli* H5, complete genome |
| CP010170 | 41 | 7 | *Escherichia* | *Escherichia coli* H6, complete genome |
| CP010171 | 41 | 7 | *Escherichia* | *Escherichia coli* H7, complete genome |
| CP010172 | 41 | 7 | *Escherichia* | *Escherichia coli* H8, complete genome |
| CP010226 | 41 | 7 | *Escherichia* | *Escherichia coli* S1, complete genome |
| CP010231 | 41 | 7 | *Escherichia* | *Escherichia coli* S30, complete genome |
| CP010237 | 41 | 7 | *Escherichia* | *Escherichia coli* S43, complete genome |
| CP010371 | 41 | 7 | *Escherichia* | *Escherichia coli* 6409, complete genome |
| CP010438 | 41 | 7 | *Escherichia* | *Escherichia coli* K-12 ER3454, complete genome |
| CP010439 | 41 | 7 | *Escherichia* | *Escherichia coli* K-12 ER3440, complete genome |
| CP010440 | 41 | 7 | *Escherichia* | *Escherichia coli* K-12 ER3476, complete genome |
| CP010441 | 41 | 7 | *Escherichia* | *Escherichia coli* K-12 ER3445, complete genome |
| CP010442 | 41 | 7 | *Escherichia* | *Escherichia coli* K-12 ER3466, complete genome |
| CP010443 | 41 | 7 | *Escherichia* | *Escherichia coli* K-12 ER3446, complete genome |
| CP010444 | 41 | 7 | *Escherichia* | *Escherichia coli* K-12 ER3475, complete genome |
| CP010445 | 41 | 7 | *Escherichia* | *Escherichia coli* K-12 ER3435, complete genome |
| CP010455 | 41 | 7 | *Escherichia* | Synthetic *Escherichia coli* C321.deltaA subrEc.y.dC.46, complete sequence |
| CP010456 | 41 | 7 | *Escherichia* | Synthetic *Escherichia coli* C321.deltaA subrEc.b.dC.12, complete sequence |
| CP010585 | 41 | 7 | *Escherichia* | *Escherichia coli* C41(DE3), complete genome |
| CP010816 | 41 | 7 | *Escherichia* | *Escherichia coli* BL21 (TaKaRa), complete genome |
| CP010876 | 41 | 7 | *Escherichia* | *Escherichia coli* MNCRE44, complete genome |
| CP011061 | 41 | 7 | *Escherichia* | *Escherichia coli* str. Sanji, complete genome |
| CP011113 | 41 | 7 | *Escherichia* | *Escherichia coli* RR1, complete genome |
| CP011124 | 41 | 7 | *Escherichia* | *Escherichia coli* USML2, complete genome |
| CP011134 | 41 | 7 | *Escherichia* | *Escherichia coli* VR50, complete genome |
| CP011320 | 41 | 7 | *Escherichia* | *Escherichia coli* SQ37, complete genome |
| CP011321 | 41 | 7 | *Escherichia* | *Escherichia coli* SQ88, complete genome |
| CP011322 | 41 | 7 | *Escherichia* | *Escherichia coli* SQ110, complete genome |
| CP011323 | 41 | 7 | *Escherichia* | *Escherichia coli* SQ171, complete genome |
| CP011324 | 41 | 7 | *Escherichia* | *Escherichia coli* SQ2203, complete genome |
| CP011342 | 41 | 7 | *Escherichia* | *Escherichia coli* K-12 GM4792 Lac+, complete genome |
| CP011343 | 41 | 7 | *Escherichia* | *Escherichia coli* K-12 GM4792 Lac-, complete genome |
| CP011495 | 41 | 7 | *Escherichia* | *Escherichia coli* NCM3722, complete genome |
| CP011915 | 41 | 7 | *Escherichia* | *Escherichia coli* PSUO2, complete genome |
| CP011938 | 41 | 7 | *Escherichia* | *Escherichia coli* C43(DE3), complete genome |
| CP012112 | 41 | 7 | *Escherichia* | *Escherichia coli* PSUO78, complete genome |
| CP012125 | 41 | 7 | *Escherichia* | *Escherichia coli* DH1Ec095, complete genome |
| CP012126 | 41 | 7 | *Escherichia* | *Escherichia coli* DH1Ec104, complete genome |
| CP012127 | 41 | 7 | *Escherichia* | *Escherichia coli* DH1Ec169, complete genome |
| CP012378 | 41 | 7 | *Escherichia* | *Escherichia coli* MEM, complete genome |
| CP012379 | 41 | 7 | *Escherichia* | *Escherichia coli* PAR, complete genome |
| CP012631 | 41 | 7 | *Escherichia* | *Escherichia coli* SF-173, complete genome |
| CP012633 | 41 | 7 | *Escherichia* | *Escherichia coli* SF-166, complete genome |
| CP012635 | 41 | 7 | *Escherichia* | *Escherichia coli* SF-088, complete genome |
| CP012868 | 41 | 7 | *Escherichia* | *Escherichia coli* str. K-12 substr. MG1655, complete genome |
| CP012869 | 41 | 7 | *Escherichia* | *Escherichia coli* K-12 subMG1655_TMP32XR1, complete genome |
| CP012870 | 41 | 7 | *Escherichia* | *Escherichia coli* K-12 subMG1655_TMP32XR2, complete genome |
| CP013025 | 41 | 7 | *Escherichia* | *Escherichia coli* 2009C-3133, complete genome |
| CP013031 | 41 | 7 | *Escherichia* | *Escherichia coli* H1827/12, complete genome |
| CP013048 | 41 | 7 | *Escherichia* | *Escherichia coli* RS76 genome |
| CP013190 | 41 | 7 | *Escherichia* | *Escherichia coli* FORC_031, complete genome |
| CP013253 | 41 | 7 | *Escherichia* | *Escherichia coli* CQSW20, complete genome |
| CP013483 | 41 | 7 | *Escherichia* | *Escherichia coli* Y5, complete genome |
| CP013658 | 41 | 7 | *Escherichia* | *Escherichia coli* uk_P46212, complete sequence |
| CP013662 | 41 | 7 | *Escherichia* | *Escherichia coli* 08-00022, complete genome |
| CP013831 | 41 | 7 | *Escherichia* | *Escherichia coli* CD306, complete genome |
| CP013835 | 41 | 7 | *Escherichia* | *Escherichia coli* JJ2434, complete genome |
| CP013837 | 41 | 7 | *Escherichia* | *Escherichia coli* JJ1897, complete genome |
| CP013952 | 41 | 7 | *Escherichia* | *Escherichia coli* HST04, complete genome |
| CP014111 | 41 | 7 | *Escherichia* | *Escherichia coli* FDAARGOS_144, complete genome |
| CP014225 | 41 | 7 | *Escherichia* | *Escherichia coli* str. K-12 substr. MG1655, complete genome |
| CP014268 | 41 | 7 | *Escherichia* | *Escherichia coli* B C2566, complete genome |
| CP014269 | 41 | 7 | *Escherichia* | *Escherichia coli* B C3029, complete genome |
| CP014270 | 41 | 7 | *Escherichia* | *Escherichia coli* K-12 DHB4, complete genome |
| CP014272 | 41 | 7 | *Escherichia* | *Escherichia coli* K-12 C3026, complete genome |
| CP014316 | 41 | 7 | *Escherichia* | *Escherichia coli* JJ1887, complete genome |
| CP014348 | 41 | 7 | *Escherichia* | *Escherichia coli* str. K-12 substr. MG1655 JW5437-1, complete genome |
| CP014488 | 41 | 7 | *Escherichia* | *Escherichia coli* G749, complete genome |
| CP014492 | 41 | 7 | *Escherichia* | *Escherichia coli* MVAST0167, complete genome |
| CP014495 | 41 | 7 | *Escherichia* | *Escherichia coli* SaT040, complete geome |
| CP014497 | 41 | 7 | *Escherichia* | *Escherichia coli* ZH193, complete genome |
| CP014522 | 41 | 7 | *Escherichia* | *Escherichia coli* ZH063, complete genome |
| CP014641 | 41 | 7 | *Escherichia* | *Escherichia coli* BLK9, complete genome |
| CP014642 | 41 | 7 | *Escherichia* | *Escherichia coli* BLK16, complete genome |
| CP014667 | 41 | 7 | *Escherichia* | *Escherichia coli* ECONIH2, complete genome |
| CP014768 | 41 | 7 | *Shigella* | Shigella sp. PAMC 28760, complete genome |
| CP015069 | 41 | 7 | *Escherichia* | *Escherichia coli* Ecol_743, complete genome |
| CP015074 | 41 | 7 | *Escherichia* | *Escherichia coli* Ecol_745, complete genome |
| CP015076 | 41 | 7 | *Escherichia* | *Escherichia coli* Ecol_448, complete genome |
| CP015085 | 41 | 7 | *Escherichia* | *Escherichia coli* O25b:H4, complete genome |
| CP015138 | 41 | 7 | *Escherichia* | *Escherichia coli* Ecol_732, complete genome |
| CP015159 | 41 | 7 | *Escherichia* | *Escherichia coli* Eco889, complete genome |
| CP015834 | 41 | 7 | *Escherichia* | *Escherichia coli* MS6198, complete genome |
| CP016007 | 41 | 7 | *Escherichia* | *Escherichia coli* NGF1, complete genome |
| CP016018 | 41 | 7 | *Escherichia* | *Escherichia coli* ER1821R, complete genome |
| CP016358 | 41 | 7 | *Escherichia* | *Escherichia coli* K-15KW01, complete genome |
| CP016404 | 41 | 7 | *Escherichia* | *Escherichia coli* 210221272, complete genome |
| CP016497 | 41 | 7 | *Escherichia* | *Escherichia coli* UPEC 26-1, complete genome |
| CP017061 | 41 | 7 | *Escherichia* | *Escherichia coli* NIVEDI C53 |
| CP017100 | 41 | 7 | *Escherichia* | *Escherichia coli* K-12 NEB 5-alpha, complete genome |
| CP017220 | 41 | 7 | *Escherichia* | *Escherichia coli* FAM21845, complete genome |
| CP017631 | 41 | 7 | *Escherichia* | *Escherichia coli* SLK172, complete genome |
| CP017844 | 41 | 7 | *Escherichia* | *Escherichia coli* FMU073332, complete genome |
| CP017979 | 41 | 7 | *Escherichia* | *Escherichia coli* str. K-12 substr. W3110 subZK126 genome |
| CP017980 | 41 | 7 | *Escherichia* | *Escherichia coli* CH611_eco genome |
| CP018103 | 41 | 7 | *Escherichia* | *Escherichia coli* MRSN352231, complete genome |
| CP018109 | 41 | 7 | *Escherichia* | *Escherichia coli* MRSN346595, complete genome |
| CP018115 | 41 | 7 | *Escherichia* | *Escherichia coli* MRSN346638, complete genome |
| CP018121 | 41 | 7 | *Escherichia* | *Escherichia coli* MRSN346355, complete genome |
| CP018206 | 41 | 7 | *Escherichia* | *Escherichia coli* MRSN346647, complete genome |
| CP018770 | 41 | 7 | *Escherichia* | *Escherichia coli* 2016C-3936C1, complete genome |
| CP018801 | 41 | 7 | *Escherichia* | *Escherichia coli* tolC-, complete genome |
| CP018962 | 41 | 7 | *Escherichia* | *Escherichia coli* Ecol_422, complete genome |
| CP018970 | 41 | 7 | *Escherichia* | *Escherichia coli* Ecol_542, complete genome |
| CP018976 | 41 | 7 | *Escherichia* | *Escherichia coli* Ecol_545, complete genome |
| CP018979 | 41 | 7 | *Escherichia* | *Escherichia coli* Ecol_656, complete genome |
| CP018983 | 41 | 7 | *Escherichia* | *Escherichia coli* Ecol_867, complete genome |
| CP018991 | 41 | 7 | *Escherichia* | *Escherichia coli* Ecol_AZ146, complete genome |
| CP018995 | 41 | 7 | *Escherichia* | *Escherichia coli* Ecol_AZ147, complete genome |
| CP019000 | 41 | 7 | *Escherichia* | *Escherichia coli* Ecol_AZ153, complete genome |
| CP019005 | 41 | 7 | *Escherichia* | *Escherichia coli* Ecol_AZ155, complete genome |
| CP019008 | 41 | 7 | *Escherichia* | *Escherichia coli* Ecol_AZ159, complete genome |
| CP019012 | 41 | 7 | *Escherichia* | *Escherichia coli* Ecol_AZ161, complete genome |
| CP019015 | 41 | 7 | *Escherichia* | *Escherichia coli* Ecol_AZ162, complete genome |
| CP019020 | 41 | 7 | *Escherichia* | *Escherichia coli* Ecol_244, complete genome |
| CP019029 | 41 | 7 | *Escherichia* | *Escherichia coli* Ecol_881 |
| CP019071 | 41 | 7 | *Escherichia* | *Escherichia coli* CRE1493, complete genome |
| CP019213 | 41 | 7 | *Escherichia* | *Escherichia coli* WCHEC050613, complete genome |
| CP019455 | 41 | 7 | *Escherichia* | *Escherichia coli* FHI_NMBU_03, complete genome |
| CP019629 | 41 | 7 | *Escherichia* | *Escherichia coli* MGY, complete genome |
| CP019777 | 41 | 7 | *Escherichia* | *Escherichia coli* NU14, complete genome |
| CP019778 | 41 | 7 | *Escherichia* | *Escherichia coli* NCTC86, complete genome |
| CP019903 | 41 | 7 | *Escherichia* | *Escherichia coli* MDR_56, complete genome |
| CP020025 | 41 | 7 | *Escherichia* | *Escherichia coli* WB61, complete genome |
| CP020058 | 41 | 7 | *Escherichia* | *Escherichia coli* AR_0061, complete genome |
| CP020116 | 41 | 7 | *Escherichia* | *Escherichia coli* AR_0104, complete genome |
| CP020368 | 41 | 7 | *Escherichia* | *Escherichia coli* BLR(DE3), complete genome |
| CP020543 | 41 | 7 | *Escherichia* | *Escherichia coli* C, complete genome |
| CP020835 | 41 | 7 | *Escherichia* | *Escherichia coli* CFSAN051542, complete genome |
| CP021175 | 41 | 7 | *Escherichia* | *Escherichia coli* 5CRE51, complete genome |
| CP021179 | 41 | 7 | *Escherichia* | *Escherichia coli* 81009, complete genome |
| CP021207 | 41 | 7 | *Escherichia* | *Escherichia coli* Z247, complete genome |
| CP021288 | 41 | 7 | *Escherichia* | *Escherichia coli* PA45B, complete genome |
| CP021454 | 41 | 7 | *Escherichia* | *Escherichia coli* H105, complete genome |
| CP021532 | 41 | 7 | *Escherichia* | *Escherichia coli* AR_0149, complete genome |
| CP021683 | 41 | 7 | *Escherichia* | *Escherichia coli* AR_0162, complete genome |
| CP021689 | 41 | 7 | *Escherichia* | *Escherichia coli* AR_0058, complete genome |
| CP021691 | 41 | 7 | *Escherichia* | *Escherichia coli* AR_0151, complete genome |
| CP021732 | 41 | 7 | *Escherichia* | *Escherichia coli* AR_0114, complete genome |
| CP021736 | 41 | 7 | *Escherichia* | *Escherichia coli* AR_0150, complete genome |

^a^:the sequence chosed from each group to analysis;^b^:the bacteria belong to the Enterobacteriaceae, but don’t know its genus.
